# An aldo-keto reductase is responsible for *Fusarium* toxin-degrading activity in a soil *Sphingomonas* strain

**DOI:** 10.1038/s41598-017-08799-w

**Published:** 2017-08-25

**Authors:** Wei-Jie He, Limin Zhang, Shu-Yuan Yi, Xue-Ling Tang, Qing-Song Yuan, Mao-Wei Guo, Ai-Bo Wu, Bo Qu, He-Ping Li, Yu-Cai Liao

**Affiliations:** 1 Molecular Biotechnology Laboratory of Triticeae Crops, Huazhong Agricultural University, Wuhan, 430070 China; 2 College of Life Science and Technology, Huazhong Agricultural University, Wuhan, 430070 China; 3 State Key Laboratory of Magnetic Resonance and Atomic and Molecular Physics, Wuhan Centre for Magnetic Resonance, Wuhan Institute of Physics and Mathematics, the Chinese Academy of Sciences, Wuhan, 430071 China; 4 College of Plant Science and Technology, Huazhong Agricultural University, Wuhan, 430070 China; 5 Key Laboratory of Food Safety Research Institute for Nutritional Sciences, Shanghai Institutes for Biological Sciences, Chinese Academy of Sciences, Shanghai, 200031 China; 6 National Center of Plant Gene Research (Wuhan), Huazhong Agricultural University, Wuhan, 430070 China

## Abstract

Degradation of toxins by microorganisms is a promising approach for detoxification of agricultural products. Here, a bacterial strain, *Sphingomonas* S3-4, that has the ability to degrade the mycotoxin deoxynivalenol (DON) was isolated from wheat fields. Incubation of *Fusarium*-infected wheat grains with S3-4 completely eliminated DON. In S3-4 DON is catabolized into compounds with no detectable phytotoxicity, 3-oxo-DON and 3-epi-DON, via two sequential reactions. Comparative analysis of genome sequences from two DON-degrading strains, S3-4 and *Devosia* D17, and one non-DON-degrading strain, *Sphingobium* S26, combined with functional screening of a S3-4 genomic BAC library led to the discovery that a novel aldo/keto reductase superfamily member, AKR18A1, is responsible for oxidation of DON into 3-oxo-DON. DON-degrading activity is completely abolished in a mutant S3-4 strain where the *AKR18A1* gene is disrupted. Recombinant AKR18A1 protein expressed in *Escherichia coli* catalyzed the reversible oxidation/reduction of DON at a wide range of pH values (7.5 to 11) and temperatures (10 to 50 °C). The S3-4 strain and recombinant AKR18A1 also catabolized zearalenone and the aldehydes glyoxal and methyglyoxal. The S3-4 strain and the *AKR18A1* gene are promising agents for the control of *Fusarium* pathogens and detoxification of mycotoxins in plants and in food/feed products.

## Introduction

Deoxynivalenol (DON) and its acetylated forms are the most abundant mycotoxins produced by *Fusarium* head blight (FHB) pathogens^1^, which infect the floret tissues of wheat, barley, maize and other small grain cereals in the field and colonize the developing grains. *Fusarium* toxins thus directly accumulate in the harvested grains, entering food/feed items such as flour and animal feed^1, 2^. DON has serious toxic effects on human and farm animals^3^ and is also phytotoxic, damaging plant tissues and acting as a virulence factor that stimulates fungal infection. Therefore, development of effective methods to control DON is essential for food/feed safety and disease management.

**Figure 1:**
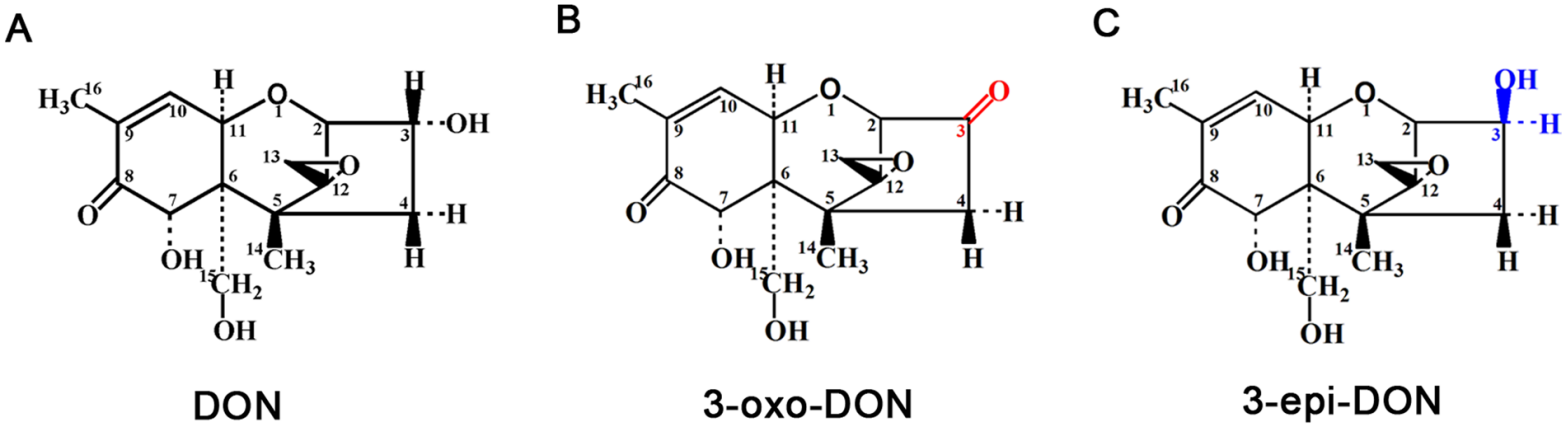
The structures of DON and its metabolites. (**A**) The structure of DON; (**B**) The structure of 3-oxo-DON; (**C**) The structure of 3-epi-DON.

Detoxification of DON by microorganisms is considered a promising method because of its efficiency, specificity, and environmental soundness^4, 5^. In the 1970s, the DON C12/13 epoxide group was identified as the primary determinant of toxicity^6^, and the de-epoxy metabolites of DON can be generated via the enzymatic reduction of the C12/13 epoxide group to adiene^5^. A bacterial strain isolated from bovine rumen fluid that de-epoxidizes DON under anaerobic conditions is used as a feed additive^6^. Aerobic bacteria capable of detoxifying DON have also been isolated and hold more promise for practical applications because cereal grains are cultivated and stored under aerobic conditions^5, 7–9^. In the 1990s, the DON C3 hydroxyl was also found to be a major determinant of toxicity^10, 11^. Based on this discovery a new detoxification pathway was proposed where DON is catabolized into 3-oxo-DON (3-keto-DON) and 3-epi-DON^5^ (Fig. 1), both of which exhibit little to no toxicity^12, 13^. Production of 3-oxo-DON occurs via oxidation of the DON C3 hydroxyl group, and a mixed culture, D107, isolated from soil could convert DON to 3-oxo-DON^14^. The soil bacteria *Nocardioides* sp. WSN05-2 was found to catabolize DON to 3-epi-DON via DON C3 epimerization^15^. Recently, a set of 13 bacteria isolated from a variety of environmental samples, such as soils and wheat leaves, were also found to efficiently transform DON into 3-epi-DON^10^. These studies have led to the speculation that DON is oxidized to 3-oxo-DON^5^, possibly by a 3-acetyl deoxynivalenol oxidase^16^, and then, 3-oxo-DON is epimerized into 3-epi-DON. However, to date no genes responsible for de-epoxidation, oxidation or epimerization of DON have been identified, and, thus, the components of these detoxification pathways remain unclear.

*Sphingomonas* is an unusual genus of the Gram-negative *alphaproteobacteria* known for their extraordinary ability to degrade recalcitrant environmental pollutants. Members of this genus are widely distributed and have been isolated from a variety of terrestrial and aquatic environments^17^. *Sphingomonas* can degrade a wide range of xenobiotic compounds such as herbicides^18^ and pesticides^19^, and thus is considered a good choice for bioremediation^20^. Recently, a *Sphingomonas* strain, KSM1, isolated from lake water was found to catabolize DON into 16-hydroxy-DON (16-HDON), which is 10-fold less phytotoxic to wheat than DON^21^. The conversion of DON to 16-HDON in this strain is mediated by a cytochrome P450 system.

Aldo-keto reductases (AKRs) have also been implicated in detoxification and are widely distributed across all organisms^22^. The AKR superfamily comprises 17 families, where members in different families share less than 40% amino acid identity. AKRs are mostly NAD(P)(H)-dependent oxidoreductases and have wide substrate spectrum. These enzymes typically catalyze the reversible reduction of a number of ketone- and/or aldehyde-containing compounds to the corresponding alcohols. Plant AKRs are involved in diverse metabolic reactions including reactive aldehyde detoxification, biosynthesis of osmolytes, secondary metabolism and membrane transport and have been exploited to generate stress tolerant transgenic plants. AKRs have also frequently been implicated in the metabolism of endogenous and exogenous toxicants^22^. AKR7A-related members from rat and human may protect against aflatoxin B1 (AFB1)-induced hepatotoxicity^23^. However, AKR superfamily members capable of detoxifying *Fusarium* toxins have not been investigated.

Here, a novel member of the AKR superfamily responsible for detoxifying *Fusarium* toxin DON was cloned from a soil *Sphingomonas* strain through comparative genome sequence analysis of bacteria with and without DON-degrading activity combined with functional screening of a *Sphingomonas* BAC library. The ability of the recombinant AKR protein to detoxify DON and other toxins was also evaluated.

## Results

### Isolation of a DON-degrading bacterial strain

An enrichment procedure was used to identify wheat field soil bacterial communities that were consistently able to degrade DON. Three antibiotics were then added to cultures with DON-degrading activity to reduce the diversity of the bacterial population. One antibiotic, streptomycin, substantially reduced population diversity without affecting the ability to degrade DON. Therefore, serial dilution was done in the presence of streptomycin to isolate single colonies with DON-degradation activity. These single colonies were then cultured in mineral salts medium (MM) containing DON, and DON levels were monitored by HPLC. One pure bacterial strain, designated S3-4, displayed stable DON-degradation activity and was selected for subsequent study.

The identity of the Gram-negative S3-4 strain was determined by sequencing its 16S rDNA gene (GenBank ID in NCBI: KY575150) and performing BLAST searches against 16S rDNA sequences retrieved from NCBI. The S3-4 rDNA sequence had the highest identity (97%) to the rDNA of *Sphingomonas leidyi* DSM 4733 (Supplementary Fig. S1). Thus, we conclude that strain S3-4 is a member of the genus *Sphingomonas*.

**Figure 2:**
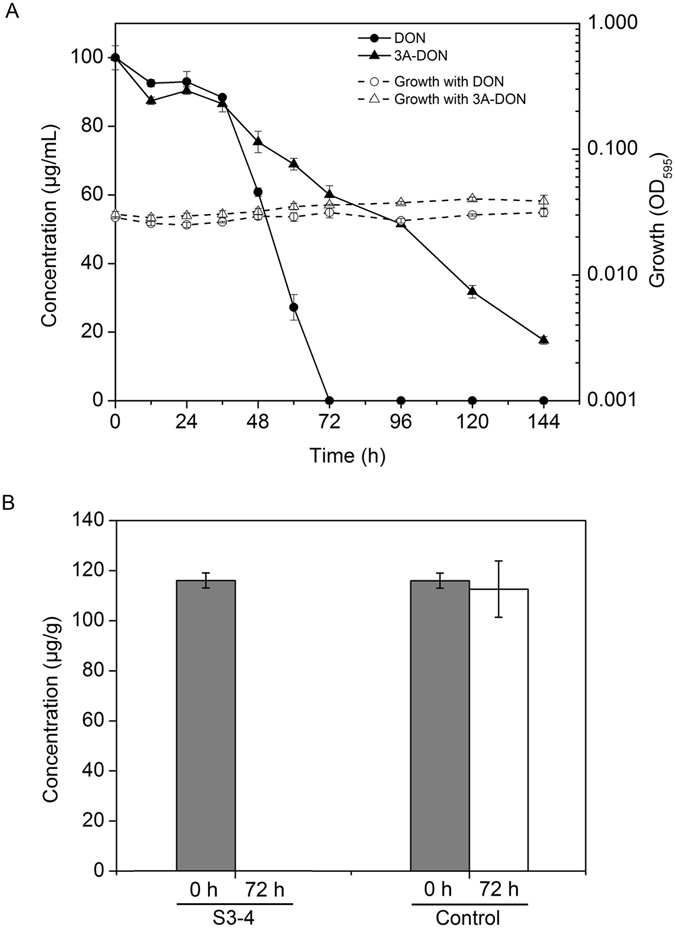
Growth and DON-degrading efficiency of strain S3-4. (**A**) Growth and degradation activity of strain S3-4 in mineral salts medium (MM) containing DON or 3A-DON (100 μg/mL). Levels of DON in the media and the amount of bacterial growth were determined every 12 h for 144 h. (**B**) Degradation activity of strain S3-4 in wheat grains contaminated with DON. Levels of DON were measured by HPLC at 0 h (gray bars) and after 72 h incubation (white bars) with or without S3-4. The values given are the means of three biological replicates. The error bars represent the standard deviation.

To investigate DON degradation activity of S3-4 in the presence of DON versus time, S3-4 was grown in MM containing DON (100 μg/mL), and changes in DON concentration were monitored every 12 hours by HPLC. Levels of DON were significantly reduced at 12 hours after inoculation (hai), were sharply decreased at 36 hai and completely absent at 72 hai (Fig. 2A). We investigated the degradation of 3-acetyl deoxynivalenol (3A-DON), which is the predominant DON-acetylated form produced by FHB pathogens in China^2^ and other countries^24^. Similar to DON, levels of 3A-DON were reduced in S3-4, but not to the same extent as DON. The number of bacterial cells remained constant during the entire culture period in the presence of DON or 3A-DON (Fig. 2A). These results indicate that S3-4 cells grew slowly and retained high DON degradation activity under oligotrophic culture conditions.

To evaluate the ability of S3-4 to degrade DON in agricultural products, wheat grains infected with *F. graminearum* and contaminated with DON were ground, incubated with S3-4 and monitored by HPLC for DON degradation. Wheat samples contained 112 μg of DON before treatment, and after incubation with S3-4 for 72 hours, no DON was detectable (Fig. 2B). In contrast, levels of DON in control samples that were incubated in MM without S3-4, were not significantly reduced after 72 hours. These results demonstrate that strain S3-4 can completely eliminate DON from wheat.

**Figure 3:**
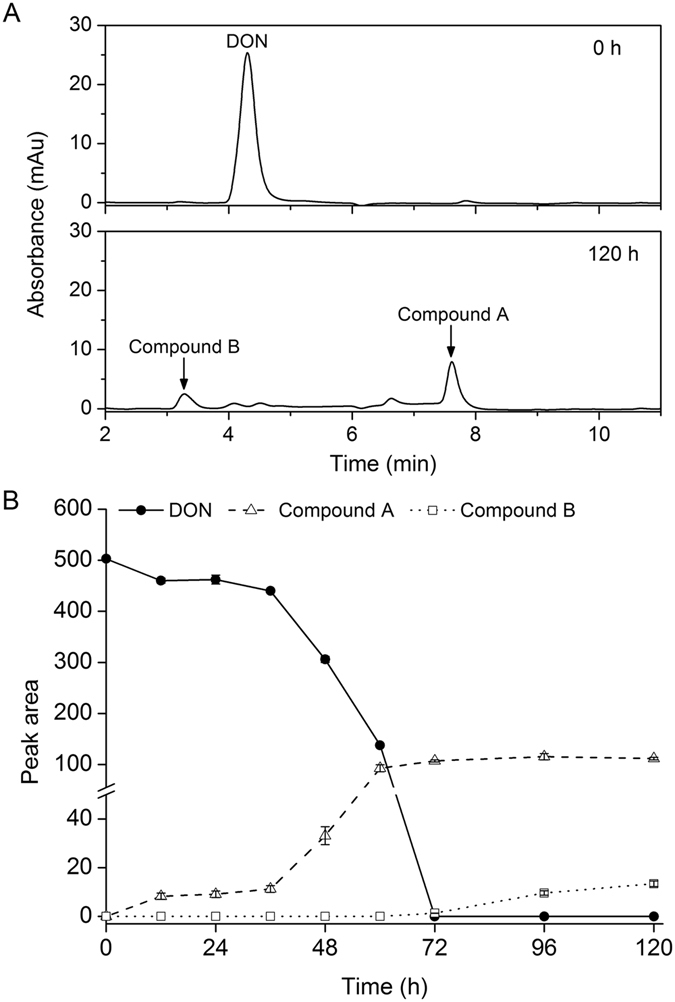
Chemical determination of DON and the products of DON catabolism in strain S3-4. (**A**) S3-4 was grown in mineral salts medium containing DON (100 μg/mL) and HPLC profiles were taken at 0 h (top panel) and 120 h (bottom panel). A representative result from three independent experiments is shown. (**B**) Depletion of DON and accumulation of its catabolites under the same conditions as in Fig. 2A during a period of 120 h. The values given are the means of three biological replicates. The error bars represent the standard deviation.

To detect the products of DON degradation, metabolites from S3-4 cultures (100 μg/mL DON) at 0 hai and 120 hai were extracted and analyzed by HPLC. At 120 hai, there were two new compounds with retention times of 7.65 min (compound A) and 3.29 min (compound B), respectively (Fig. 3A). These compounds were produced at different times after inoculation with DON; significant levels of compound A accumulated at 12 hai, then levels sharply increased at 60 hai, and remained high at 120 hai. In contrast, compound B was not detectable until 72 hai and then levels slightly increased (Fig. 3B). In addition, based on the peak areas from the HPLC profiles, levels of compound A were 80-fold (72 hai) and 8-fold higher (120 hai), respectively, than compound B. These results indicate that strain S3-4 sequentially catabolizes DON into two compounds, with compound A being the first and predominant product.

### Identification of DON catabolites by gas-chromatograph/mass spectroscopy and nuclear magnetic resonance

**Figure 4:**
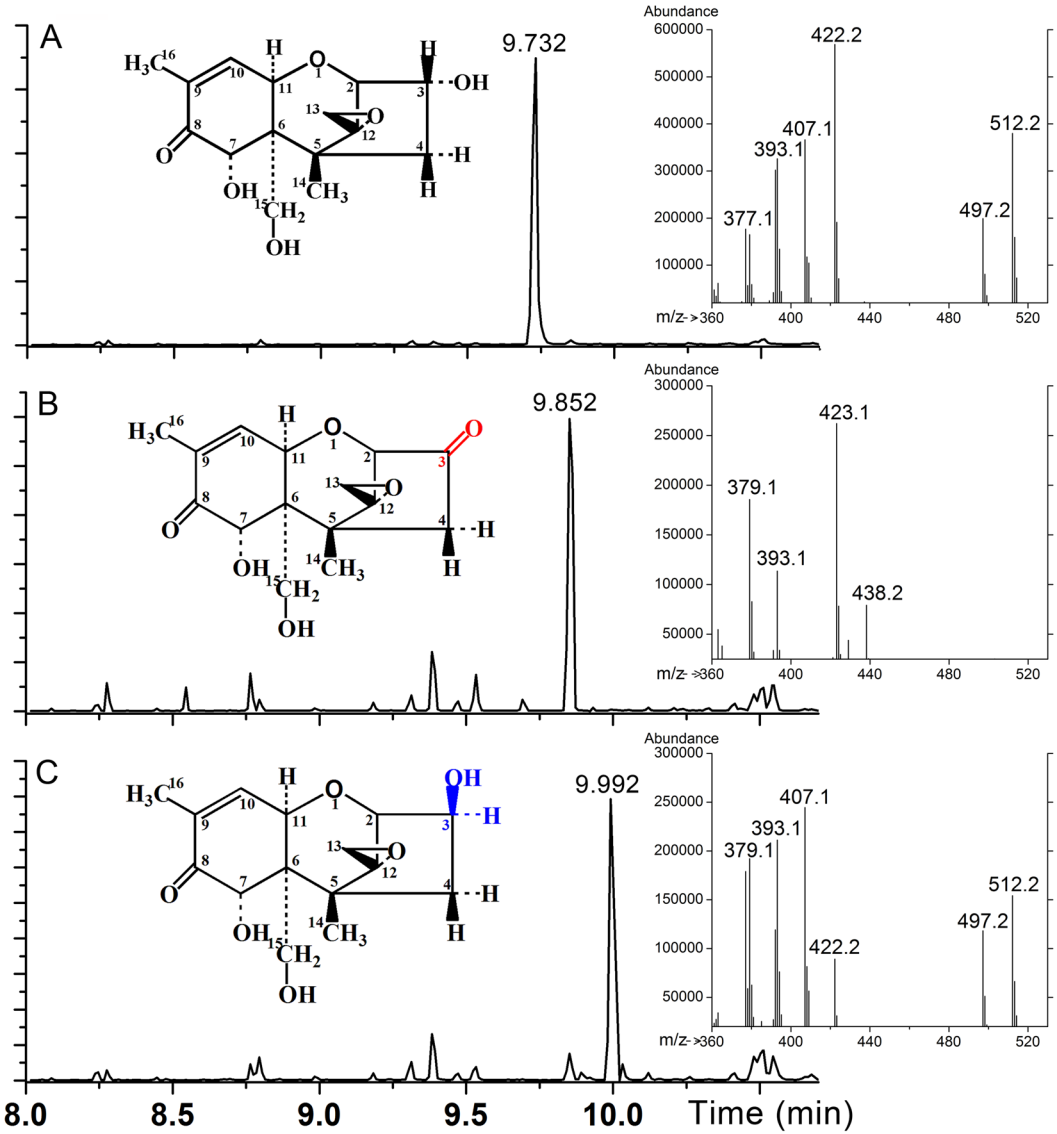
Gas chromatography (GC) and mass spectrometry (MS) of DON and its metabolites. (**A**) Total ion chromatogram of DON. Mass spectra for peaks representing DON are shown in the right insert. (**B**) Total ion chromatogram of compound A. Mass spectra for peaks representing compound A are shown in the right insert. (**C**) Total ion chromatogram of compound B. Mass spectra for peaks representing compound B are shown in the right insert. The structures of DON and its metabolites are shown in the left inserts.

Compounds A and B were purified by semi-preparative HPLC and along with DON were subjected to analysis by gas-chromatograph/mass spectroscopy (GC/MS) to determine their identities. Derivatized DON and its two catabolites (Supplementary Fig. S2) each had a distinct retention time (Fig. 4), confirming that they are structurally distinct. Molecular mass for each derivatized compound was determined from MS: DON is 512.2 Da (the molecular mass of DON is 296 Da, and the molecular mass of the three trimethylsilyl groups used for derivatization is 216 Da), compound A is 438.2 Da (the difference from derivatized DON was 74, and the molecular mass of the trimethysilyl group is 72, leaving a 2 dalton difference between DON and compound A), and compound B is 512.2 Da (the same as DON).

These compounds were further characterized by ^1^H nuclear magnetic resonance (NMR) spectroscopy (Supplementary Fig. S3). All NMR assignments for compounds A and B were compared with previously published NMR data for 3-oxo-DON and 3-epi-DON^11, 15^. The NMR experimental parameters, including ^1^H chemical shifts, multiplicity and J-coupling constants, of compounds A and B closely match those of 3-oxo-DON and 3-epi-DON, respectively (Supplementary Table S1). GC/MS (Fig. 4) and NMR results strongly indicate that compounds A and B are 3-oxo-DON and 3-epi-DON, respectively.

### Reduced impact of 3-oxo-DON and 3-epi-DON on wheat seedlings

**Figure 5:**
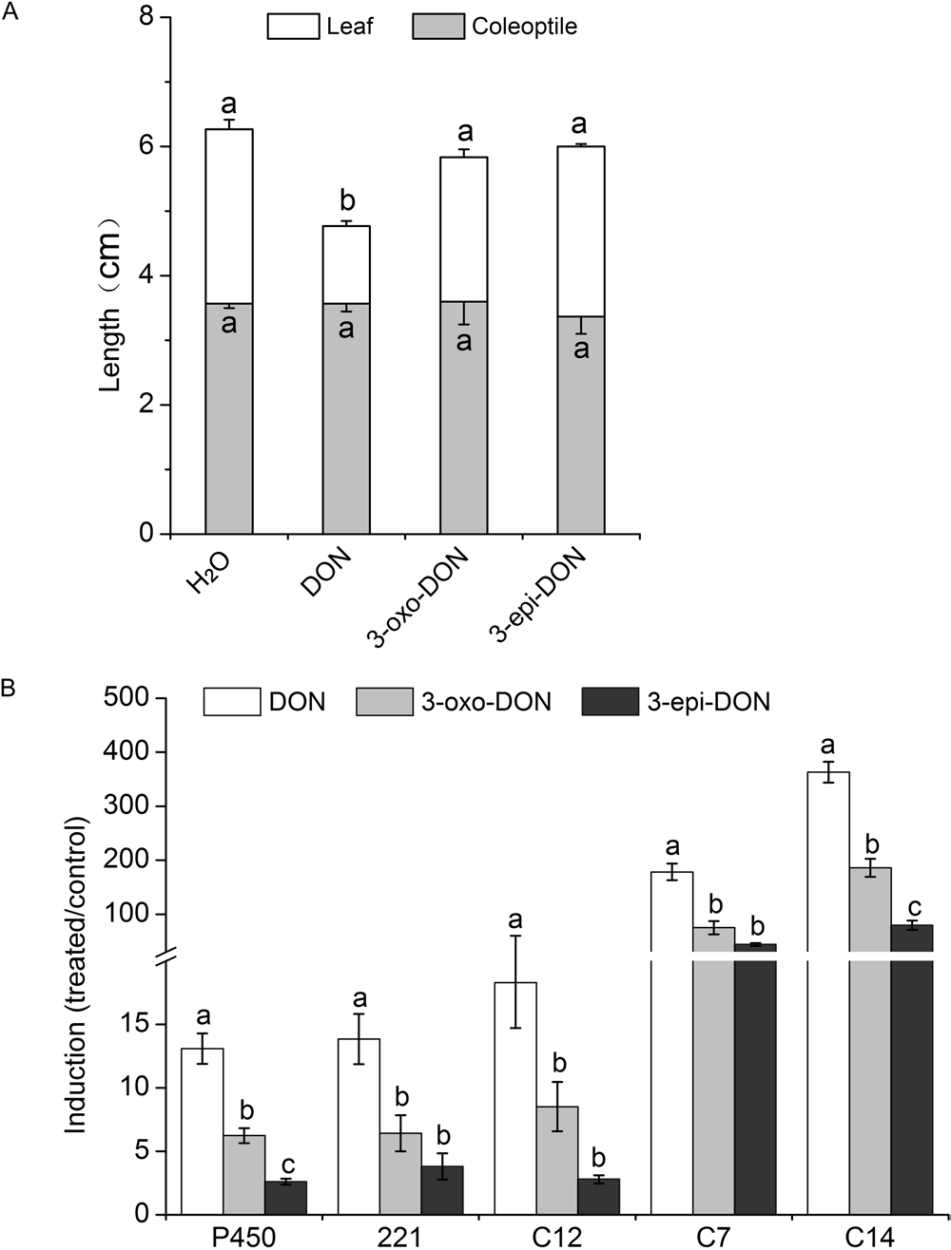
Toxicity of DON, 3-oxo-DON and 3-epi-DON to wheat seedlings. (**A**) The lengths of wheat leaves (white bars) and coleoptiles (gray bars) 24 hours after inoculation with DON, 3-oxo-DON, 3-epi-DON and H_2_O (control). (**B**) qRT-PCR determination of the expression levels of DON-responsive genes in the wheat samples from A. The levels of gene transcripts are calculated relative to the levels in the water inoculation sample.

To determine whether the two DON-degradation compounds have reduced phytotoxicity, wheat coleoptiles were inoculated with 100 μg/mL of DON, 3-oxo-DON and 3-epi-DON, respectively, and the lengths of wheat leaves and coleoptiles were measured 24 hai. As shown in Fig. 5A, there were no significant differences in coleoptile length between the four treatments. However, DON inoculation significantly reduced wheat leaf length (56%) compared with the water control, whereas 3-oxo-DON or 3-epi-DON inoculation did not significantly affect leaf length. Thus, in wheat, both 3-oxo-DON and 3-epi-DON have no or undetectable phytotoxicity.

### 3-oxo-DON and 3-epi-DON have reduced impact on the expression of DON-responsive genes in wheat seedlings

We next evaluated the effect of DON, 3-oxo-DON and 3-epi-DON on DON-responsive gene expression in wheat seedlings at 24 hai. Five wheat marker genes known to be induced by DON were assayed by qRT-PCR. Transcript levels for all five genes were significantly lower in 3-oxo-DON or 3-epi-DON-treated seedlings than in seedlings treated with DON, with reduction in transcript levels ranging from 2.0- to 6.6-fold (Fig. 5B). These results indicate the impact of 3-oxo-DON and 3-epi-DON on gene expression is significantly reduced compared with DON.

### Identification of oxidoreductase genes by comparative genomic sequence analysis

To perform comparative genome analysis with other bacteria, the S3-4 genome was sequenced. The S3-4 genome comprises three scaffolds (3,153,523 bp, 1,058,422 bp and 397,147 bp in length, respectively) and two plasmids (155,520 bp and 12,966 bp in length, respectively), with a total genome size of 4.7 Mb. There are 4,593 predicted genes, including two 5 S rRNA, two 23 S rRNA, two 16 S rRNA, and 50 tRNA genes.

**Figure 6:**
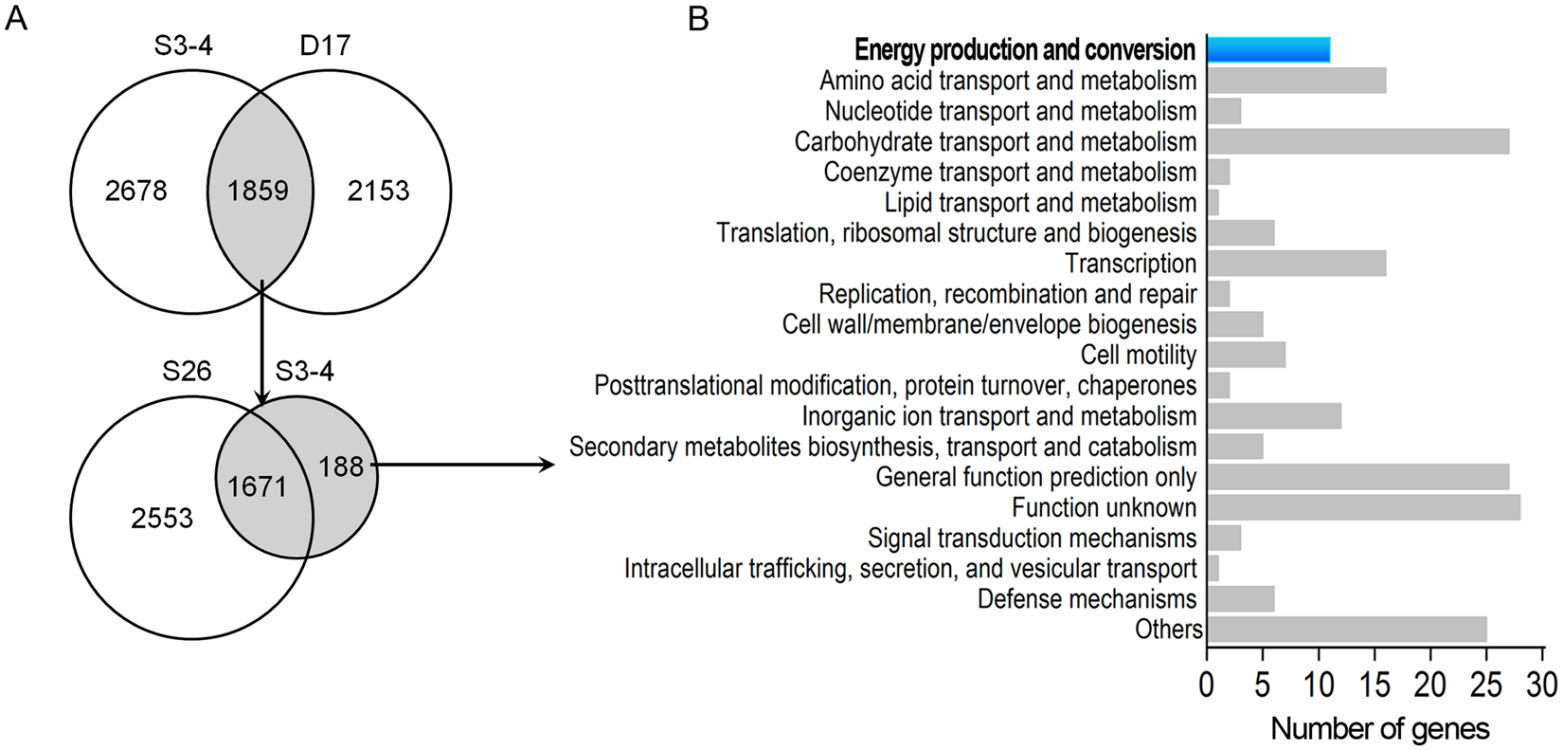
Candidate genes for oxidation revealed by comparative genome sequence analysis. (**A**) The Venn diagram shows comparisons of gene numbers in the *Sphingomonas* sp. strain S3-4 (S3-4), *Devosia* sp. strain 17-2-E-8 (D17) and *Sphingobium japonicum* UT26 (S26). Overlapping regions represent genes common to different genomes. (**B**) Functional classification of the genes unique to strain S3-4 according to the COG database.

The annotated protein sequence of S3-4 was compared with the genome sequences of *Devosia* sp. 17-2-E-8 (D17), which is able to degrade DON^25^. Comparative analysis revealed that 1,859 protein-coding genes are conserved between D17 and S3-4 (Fig. 6A). A second BLASTp search was conducted to compare these 1,859 proteins against the genome of *Sphingobium japonicum* UT26 (S26), which is a close relative of S3-4 but does not have DON-degrading activity^21^. This comparison revealed that 188 of the 1,895 protein-coding genes conserved between D17 and S3-4 are not present in S26 (Fig. 6A). These 188 genes unique to the DON-degrading strains were subsequently used for identification of candidate genes responsible for DON-degradation in the S3-4 strain.

Functional annotation was done using the COG (Clusters of Orthologous Groups) database. Of the 188 unique genes, 163 could be classified into 19 functional categories (Fig. 6B). Eleven genes in the class of energy production and conversion were selected for further analysis. This class was chosen because genes responsible for energy production and conversion likely play an essential role in allowing S3-4 to grow in MM media containing DON as the only carbon source by metabolizing DON to generate energy for bacterial growth. Because the transformation of DON into 3-oxo-DON is actually the oxidation of alcohol, which may be catalyzed by a dehydrogenase, 5 of the 11 genes that encode predicted oxidoreductases (related to aryl-alcohol dehydrogenases) were considered potential DON degradation candidate genes.

### Identification of an oxidoreductase gene by functionally screening a BAC library

**Figure 7:**
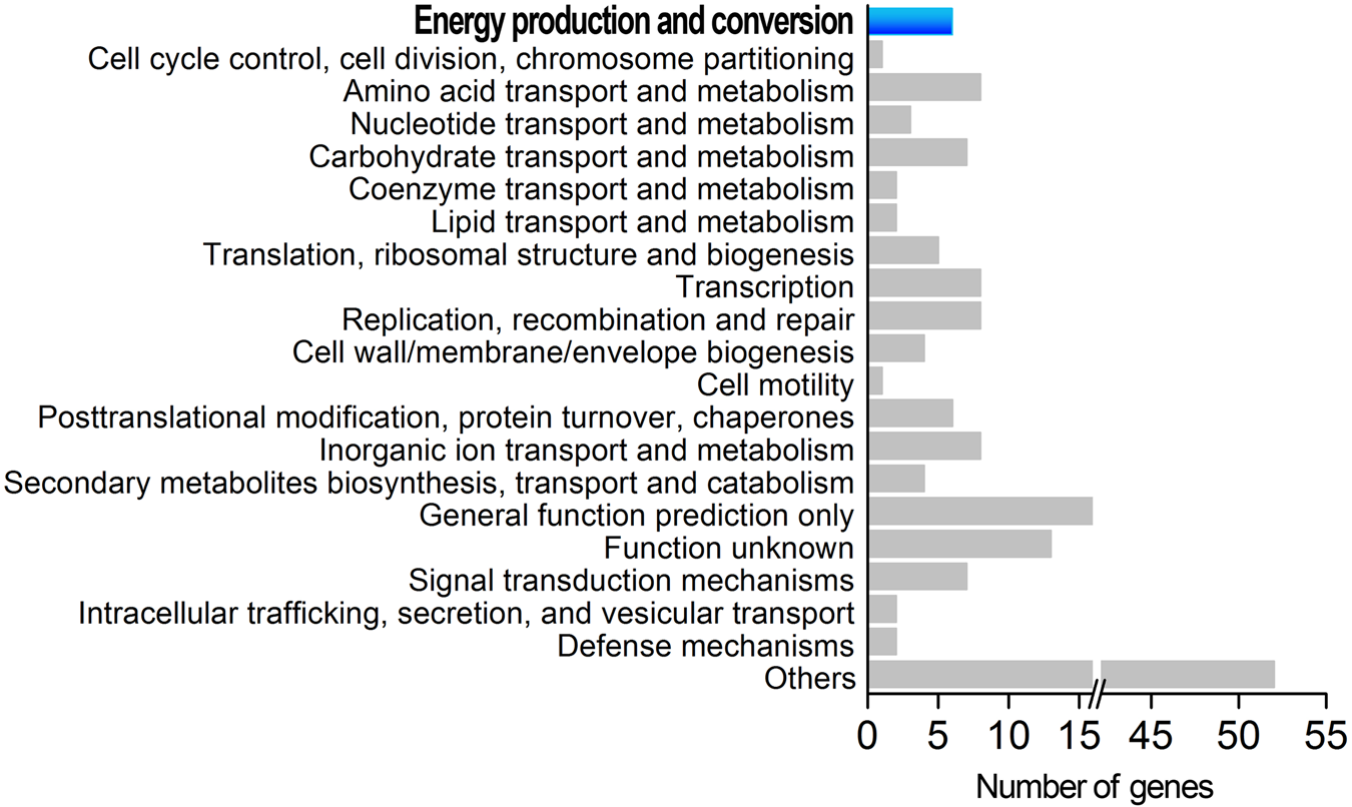
Functional classification of genes in one positive BAC clone isolated by functional screening. Genes functions were classified according to the COG database.

To identify the gene responsible for DON-degradation, a S3-4 BAC library was screened for enzymatic activity. This library consists of 2,304 clones that were arrayed in six 384-well plates. Analysis of 30 random BAC clones showed that the library had an average insert size of 120 kb with a size range from 20 to 227.5 kb and an empty-vector rate of lower than 5% (Supplementary Fig. S4). The genome coverage of this library is estimated to be more than 50-fold (based on a genome size of 4.7 Mb). When this BAC library was screened for DON-degrading activity, one positive clone was identified. The insert of this clone was 148 kb in length and contained 152 genes. These genes can be classified into 20 categories based on COG database annotation, with 6 genes belonging to the energy production and conversion category (Fig. 7). One of these genes, which was also identified as a gene unique to S3-4 and D17, encodes a predicted oxidoreductase, designated A1 (GenBank accession number: MF314460). This oxidoreductase gene is one of the five predicted oxidoreductases that were identified by comparative genomic sequence analysis (Fig. 6B) and was then further characterized.

### A new aldo/keto reductase family member AKR18 A1 is responsible for DON-degradation

Alignment of the amino acid sequences of the S3-4 A1 protein and aldo/keto reductases retrieved from BLAST searches showed that the A1 protein contains typical AKR signature motifs, such as an (α/β)_8_-barrel motif and catalytic tetrad (Asp-57, Tyr-62, Lys-90, and His-131), which are conserved among aldo-keto reductases (Supplementary Fig. S5). The AKR superfamily comprises 17 families, with more than 190 members (as of July 2015, http://www.med.upenn.edu/akr/). No AKR protein has more than 40% amino acid sequence identity to the A1 protein. A phylogenetic tree was generated based on amino acid sequence alignments of all 24 annotated AKRs from bacteria (http://www.med.upenn.edu/akr/) and the A1 protein. Based on this tree, A1 is most closely related to AKR12, but only has 37%, 36% and 35% identity to AKR12A1, AKR12B1 and AKR12C1, respectively (Supplementary Fig. S6). The nomenclature criterion for the AKR family is >40% identity^26^; therefore, these results led us to propose a new AKR family, family 18, and A1, designated AKR18A1, as the first member of this family.

### Enzymatic properties of recombinant AKR18A1 expressed from *E. coli*

**Figure 8:**
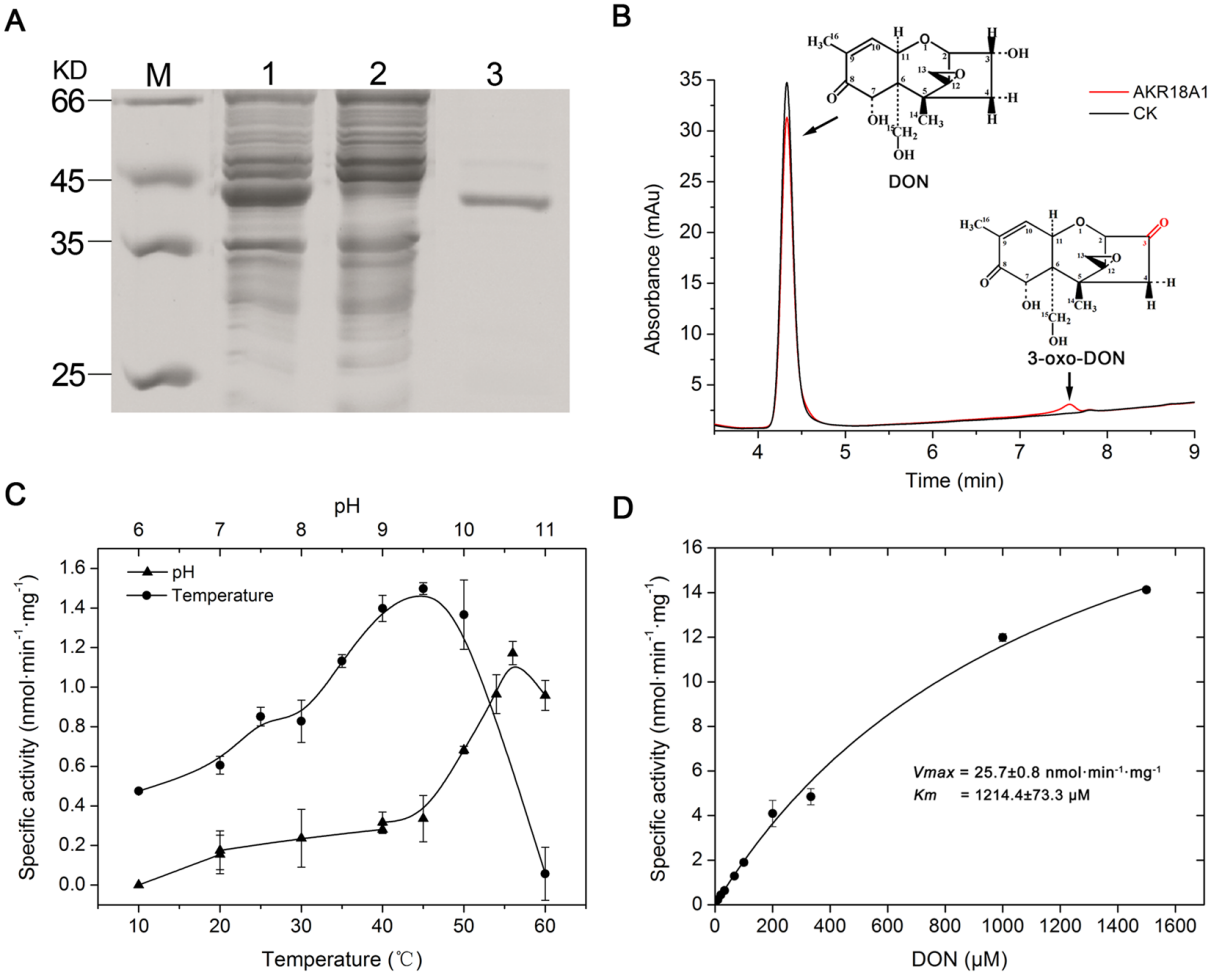
Expression and functional characterization of AKR18A1. (**A**) SDS-PAGE analysis of purified AKR18A1. Lane 1, crude extract of recombinant *E. coli* BL21 cells with IPTG induction; Lane 2, soluble fraction of crude extract of recombinant *E. coli* BL21 cells; Lane 3, purified recombinant AKR18A1. (**B**) HPLC profiles of 3-oxo-DON, the product of *in vitro* DON oxidation by recombinant AKR18A1, in the presence of cofactor NADP^+^. Insert: structure of DON and 3-oxo-DON. (**C**) Effect of pH (triangles), and temperature (circles) on recombinant AKR18A1 activity in the presence of NADP^+^. (**D**) Kinetic parameters of recombinant AKR18A1 activity in the presence of NADP^+^. The values given are the mean of three biological replicates. The error bars represent the standard deviation.

AKR18A1 was expressed in *E. coli* BL21, purified by affinity-chromatography and assayed for its DON-degrading activity and enzymatic properties. This recombinant protein had the expected size based on SDS-polyacrylamide gel electrophoresis (Fig. 8A) and was used in all enzymatic activity assays. The recombinant AKR18A1 protein oxidized DON to form 3-oxo-DON in the presence of cofactor NADP^+^ (Fig. 8B), whereas no activity was seen in the presence of NAD^+^ (data not shown), indicating that NADP^+^ is essential for DON-degradation activity. The AKR18A1 protein catalyzed a reverse reaction of 3-oxo-DON into DON in the presence of the cofactor NADH (Supplementary Fig. S7), whereas no activity was seen in the presence of NADPH (data not shown).

Recombinant AKR18A1 had the highest activity at pH 10.6 and 45 °C, and activity was maintained over a wide range of pH values (from 7 to 11) and temperatures (from 10 to 50 °C), implying high stability and activity under different conditions (Fig. 8C). Km and Vmax values for AKR18A1 were 1214.4 ± 73.3 μM and 25.7 ± 0.8 nmol·min^−1^·mg^−1^ protein, respectively (Fig. 8D), and for the cofactor NADP^+^ in the oxidation reaction, the Km and Vmax values were 480 ± 70 μM and 53.7 ± 2.4 nmol·min^−1^·mg^−1^ protein, respectively. Regarding reversible reduction reaction from 3-oxo-DON into DON, the Km and Vmax values for AKR18A1 were 547.1 ± 121.4 μM and 176.1 ± 19.5 nmol·min^−1^·mg^−1^ protein, respectively, while for the cofactor NADH the Km and Vmax were 78 ± 28 mM and 147.5 ± 5.2 nmol·min^−1^·mg^−1^ protein, respectively.

### Disruption of AKR18A1 in *Sphingomonas* sp. strain S3-4

To further verify that AKR18A1 is responsible for DON-oxidation in strain S3-4, its coding sequence was disrupted by gene replacement with a disruption plasmid pK18*mobsacB*, generating an isogenic *Sphingomonas* strain, *Δ*akr18a1, that differs from the strain S3-4 only in a single gene, *AKR18A1*. The specific disruption of *AKR18A1* was confirmed by PCR (Supplementary Fig. S8). The mutant *Δ*akr18a1 and wild-type (WT) S3-4 strains had similar growth patterns in nutrient broth (NB) medium. When WT S3-4 was cultured in MM supplemented with 100 μg/mL DON, DON was substantially degraded at 36 hai and completely degraded at 72 hai. However, when the mutant strain *Δ*akr18a1 was cultured under the same conditions, DON was not degraded at all during the entire culture period while the number of cells remained constant (Supplementary Fig. S9). The abolishment of DON-degrading activity in *Δ*akr18a1 demonstrates that *AKR18A1* is the only gene responsible for oxidation of DON in S3-4.

### Catabolism of zearalenone by strain S3-4 and recombinant protein AKR18A1

To determine whether AKR18A1 can target the ketone group of another *Fusarium* mycotoxin, zearalenone (ZEN), the ZEN-degrading activities of S3-4 and recombinant AKR18A1 protein were assayed. When cofactor NADH was present, recombinant AKR18A1 protein catabolized ZEN into α-zearalenol (α-ZOL) and β-zearalenol (β-ZOL) (Supplementary Fig. S10A). However, when NADPH was used as a cofactor, no catabolizing activity was seen (Supplementary Fig. S10B). Furthermore, in the presence of NADP^+^, α-ZOL and β-ZOL could be converted to ZEN (Supplementary Fig. S10C and D). Strain S3-4 was able to catabolize the same reactions (Supplementary Fig. S10E and F). Thus, S3-4 is able to catabolize ZEN, α-ZOL and β-ZOL, and the catabolic activity of the AKR18A1 protein is dependent on cofactors.

### Degradation of aldehydes by recombinant AKR18A1 *in vitro* and in *E. coli*

To determine if AKR18A1 protein degrades aldehydes, an *E. coli* strain BL21 expressing the *AKR18A1* gene was grown in the presence of glyoxal (GO) and methyglyoxal (MG). This strain was highly tolerant to GO and MG, whereas a BL21 strain containing a control plasmid pET-22b was sensitive to GO and MG (Supplementary Fig. S11A and B). In addition, the purified recombinant AKR18A1 protein could efficiently degrade GO and MG (Supplementary Fig. S11C and D). These results demonstrate that AKR18A1 can catabolize aldehyde compounds.

## Discussion

**Figure 9:**
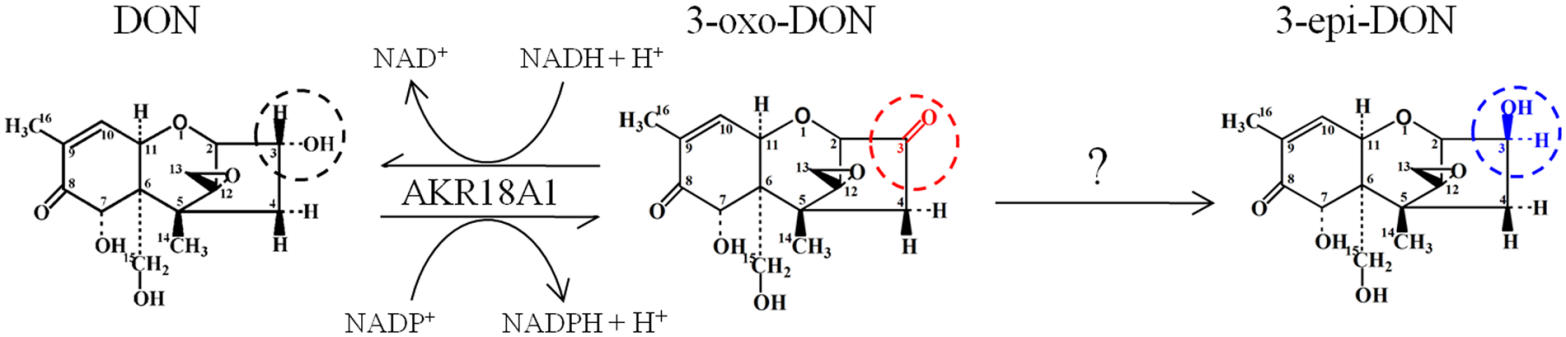
Proposed pathway for the transformation of DON into 3-oxo-DON and 3-epi-DON in strain S3-4.

In this study function-based screening and comparative analysis were used to isolate a DON-detoxifying bacterial strain, S3-4, and the gene responsible for catabolic activity, *AKR18A1*. Biotransformation of DON into two different compounds, 3-oxo-DON and 3-epi-DON, in the same strain provides direct evidence that DON degradation occurs via sequential reactions. To date, only two bacterial strains, one *Devosia* ^10, 11^ and one mixed culture^14^, are known to oxidize DON into 3-oxo-DON, whereas several *Devosia* and *Nocardioides* strains have been shown to epimerize DON into 3-epi-DON^10, 15, 27^. However, there is no a single strain that can catabolize DON into both compounds. It has been proposed that DON is first oxidized to 3-oxo-DON and then converted to 3-epi-DON^5^. In the present study we demonstrate that these reactions are sequential (Fig. 9). First, AKR18A1 catalyzes the reversible oxidation/reduction of DON to 3-oxo-DON and second, 3-oxo-DON is converted to 3-epi-DON by an unknown enzyme. In strain S3-4 there is much higher oxidation activity than epimerization activity, but the mechanistic basis for this differential activity remains unknown.

The S3-4 AKR18A1 protein contains conserved domains typical of AKR family members but is less than 40% identical to other AKR proteins. Therefore AKR18A1 represents a new member of the AKR superfamily. The AKR18A1 protein carries conserved sequences that have been identified in sequence alignments and structural analysis of AKRs. For instance, the residues of the cofactor-binding pocket across all the AKRs are strictly conserved (Asp, Asn, Gln, and Ser)^26^, and these residues are all found in the AKR18A1 sequence (Asp-50, Asn-162, Gln-187, Ser-268) (Supplementary Fig. S5). The substrate-binding pocket for the AKRs is formed by the carboxyl-terminal regions of the central β-strands where there is a conserved catalytic tetrad of Tyr, Lys, His, Asp^26^. This catalytic tetrad is also present in AKR18A1 (Supplementary Fig. S5).

The AKR18A1 protein appears to have favored catalytic direction towards the reverse reduction reaction of 3-oxo-DON into DON *in vitro* assays, whereas in the S3-4 strain, DON is completely converted into 3-oxo-DON and 3-epi-DON (Fig. 2B). This discrepancy between recombinant the AKR18A1 protein and the S3-4 strain may relate to the presence of other proteins in the S3-4 strain that may catalyze the 3-oxo-DON to 3-epi-DON; it has been speculated that during microbial conversion from DON into 3-oxo-DON and 3-epi-DON the coupling of the transformation to another pathway may drives it away from equilibrium^26^.

In addition to degrading DON, AKR18A1 catalyzes the reduction of ZEN into α-ZOL and β-ZOL (Supplementary Fig. S10), as well as the reduction of the aldehydes, GO and MG (Supplementary Fig. S11). Thus, AKR18A1 can catalyze the reduction of a wide range of ketones and/or aldehyde-containing compounds and the oxidation of alcohols. It is likely that AKR18A1 could reduce aflatoxin dialdehyde to mono and bis-alcohols, as has been demonstrated for the AKR superfamily members AKR7A2 and 7A3. This reaction can prevent aldehydes from forming Schiff’s bases with lysine residues and thus reduce the hepatotoxicity of aflatoxins^22^. Therefore, the AKR18A1 protein could be used for degrading different toxins.

The lower degrading activity for 3A-DON than for DON (Fig. 2) suggests that an additional reaction to remove acetyl group at the C3 position by unknown enzymes is required before oxidation of DON into 3-oxo-DON. Supporting this view, recombinant AKR18A1 enzyme did not oxidize 3A-DON into 3-oxo-DON (data not shown). Indeed, when strain S3-4 was grown in the presence of 3A-DON, HPLC assays revealed that DON appeared first, followed by 3-oxo-DON (data not shown). The acetyl group at the C3 position may thus interfere with oxidation activity. In addition, neither the AKR18A1 enzyme or strain S3-4 degraded nivalenol (NIV) (data not shown), another mycotoxin produced by some species of *F. graminearum*. NIV and DON are structurally similar sesquiterpenes bearing C3 hydroxyl groups. The only difference between them is a C4 hydroxyl group in NIV and a C4 hydrogen group in DON; the NIV C4 hydroxyl group may reduce the accessibility of the C3 hydroxyl group to AKR18A1. This finding is consistent with a previous report that UDP-glucosyltransferase can act on DON but not NIV^28^. Thus, although AKRs are promiscuous enzymes, individual AKRs have unique substrate specificities. The strain S3-4 was isolated from a screen where DON was the sole carbon source, and it is conceivable that strains capable of detoxifying NIV may also be isolated if NIV is used as the carbon source for screening.

The toxicity of 3-oxo-DON is much lower than DON, and in mouse has only one tenth the immunosuppressive toxicity of DON^11^. Recent studies have reported that 3-epi-DON has almost no toxicity^13^. In the present study both 3-oxo-DON and 3-epi-DON were not toxic to wheat seedlings (Fig. 5). DON in wheat grains at levels 112-fold higher than the mycotoxin limit for consumption (1 μg/g) was completely eliminated by strain S3-4 within 72 h (Fig. 2), indicating the great practical potential of S3-4 in decontaminating food/feed stuff. A large-scale culture of this strain via fermentation and its application for detoxification of DON-contaminated food/feed products are in progress. The recombinant AKR18A1 protein can also be used to detoxify DON. Moreover, because DON is a virulence factor that is required for the spread of plant FHB pathogens^1^, the *AKR18A1* gene can potentially be used to control plant diseases caused by DON-producing pathogens. The improvement of plant resistance against *Fusarium* pathogens and mycotoxins is a major challenge because most cultivars currently grown are susceptible to this disease and no highly resistant germplasm is available. Thus the identification of new genes to control *Fusarium* mycotoxins, such as *AKR18A1*, is particularly important.

## Conclusion

In the present study a bacterial strain, S3-4, capable of detoxifying *Fusarium* toxins was isolated and the gene responsible for detoxification, *AKR18A1*, was cloned. Both the S3-4 strain and the recombinant AKR18A1 protein could be used as detoxifying agents to control FHB pathogens and to reduce mycotoxin levels in food and feed products. These results serve as the basis for future isolation of novel genes that detoxify mycotoxins and dissection of the complete pathway for degradation of *Fusarium* toxins via oxidation and epimerization.

## Materials and Methods

### Soil, chemicals, media and plants

DON-biotransforming microorganisms were isolated from soil samples collected from a wheat field at Huazhong Agricultural University in Wuhan, China, where there are frequent FHB epidemics. DON was purified from *F. graminearum* 52701^2^ as previously described^9^. Standard chemicals such as DON, 3A-DON, NIV, ZEN, α-ZOL, β-ZOL, GO and MG were purchased from SIGMA (St Louis, MO, USA). MM supplemented with DON as a carbon source was used to screen microbial cultures for DON biotransformation capability^15^. Nutrient agar (NA) and 10-fold-diluted NA plates were used to isolate single colonies. Wheat (*Triticum aestivum*) cv. Sumai 3 was used for seedling inoculation.

### Enrichment of bacterial cultures with DON degradation activity

Soil samples (5 g) were suspended in sterile water (50 mL), and after the soil settled, 250 μL of the resulting supernatants were incubated in 2.5 mL of MM supplemented with 50 μg DON per mL as previously described^9^. The cultures were incubated at 28 °C for 7 days with shaking at 220 rpm. 100 μL of each culture was then transferred to fresh MM medium containing DON, followed by 7 days of incubation under the same conditions. This procedure was repeated seven times. Levels of DON in the cultures were monitored by HPLC as described below. Cultures with decreased DON concentrations were selected.

### Isolation of a DON-degrading strain from a bacterial community

The cultures obtained from enrichment were treated with antibiotics and serially diluted in water to reduce the diversity of the microbial population without affecting the ability of the community to degrade DON. The antibiotics used were: ampicillin (100 μg/mL), rifampicin (100 μg/mL) and streptomycin (100 μg/mL). Aliquots (0.1 mL) of cultures were spread onto plates containing 10-fold-diluted NA and incubated at 28 °C for 5 days. Randomly selected bacterial colonies were tested for DON-degradation activity, which was monitored by HPLC as described below. Strains with DON-degrading activity were stored in 25% glycerol at −80 °C until use.

### Taxonomic characterization of the isolated bacterial strain

The strain S3-4 was subjected to Gram staining, morphological observation, and 16 S rDNA gene analysis. The partial 16 S rDNA gene was amplified by PCR using the primers shown in Table S2 and sequenced to obtain 1,445 bp of 16 S rDNA sequence. Sequences similar to the S3-4 16 S rDNA were identified using BLAST (National Center for Biotechnology Information, http://www.ncbi.nlm.nih.gov). Maximum likelihood (ML) phylogenetic trees of the 16 S rDNA sequences from 26 bacterial species were constructed using MEGA 6 software. All sequences were retrieved from NCBI (http://www.ncbi.nlm.nih.gov).

### Extraction and detection of trichothecenes toxins and metabolites

DON and its metabolized products were extracted and measured as previously described^15^ using HPLC^9^. DON-biotransformed products were purified by semi-preparative HPLC. The purified compounds A and B were analyzed by GC/MS and NMR.

### GC/MS and NMR analysis

GC/MS analysis of the purified compounds A and B was performed using an Agilent 7890 A gas chromatograph equipped with an Agilent 5975 C mass spectroscopy detector as previously described^9^. MS data were collected in full scan mode at mass range m/z 100 to 600.

^1^H NMR spectra of both compounds were acquired at 298 K on a Bruker Avance III 600 MHz spectrometer equipped with a Bruker inverse cryogenic probe (Bruker Biospin, Germany). A standard one-dimensional NMR spectrum was recorded for each sample dissolved in deuterated chloroform by employing the first increment of NOESY pulse sequence (NOESYPR1D) and 90° pulse set to approximately 10 μs. A total of 64 scans were collected with 32 K data points for each spectrum with a spectral width of 20 ppm and recycle delay of 2 s. All free induction decays were multiplied by an exponential function with a 1 Hz line broadening factor prior to Fourier transformation. ^1^H NMR chemical shifts were determined with respect to the residual chloroform and referred to sodium 3-trimethylsilyl [2,2,3,3-d_4_] propionate (TSP-d_4_). ^1^H NMR resonances of the metabolites were assigned to specific protons based on previous publications^11, 15^.

### Assay of S3-4 DON-degrading activity in wheat grains contaminated with DON

Wheat grains from spikes that were infected with FHB pathogens in the field at Huazhong Agricultural University, Wuhan, China were ground, passed through a 40-mesh screen and then autoclaved at 121 °C for 18 min. Five grams of autoclaved wheat sample was mixed with 20 mL MM in a 50-mL flask. The mixture was inoculated with 5 mL bacterial suspension of *Sphingomonas* sp. S3-4 (1 × 10^10^ CFU mL^-1^) or MM and incubated at 28 °C for 72 h in an aerobic chamber. The samples were freeze-dried. DON was extracted and assayed by HPLC as described above.

### Inoculation of wheat seedlings with DON and quantitative real-time PCR

Wheat seedlings were inoculated with DON and its metabolites as described^29^ and seedling lengths were scored 24 h after inoculation. Total RNA from wheat seedlings that were harvested 24 hai were used for reverse transcription and quantitative real-time PCR as previously described^29^. Five genes, P450 (cytochrome P450), 221 (Efflux family protein), C12 (Glutathione S-transferase), C7 (methionyl-tRNA synthetase) and C14 (Putative kinase), which are induced in wheat in response to DON^30^, were quantitatively assayed. Primers are listed in Table S2.

### BAC library construction

A BAC vector was prepared with *Hin*d III (Fermentas, MA, USA) from the high-copy composite vector pHZAUBAC1 and was used for construction of a BAC library with *Hin*d III digested genomic DNA isolated from strain S3-4 as previously described^31^. One mL overnight culture for each BAC clone was suspended in 1 mL MM containing DON (20 μg/mL). After incubation for 5 days at 37 °C, metabolites were extracted and analyzed by HPLC. The positive clone was sequenced at both ends by BGI (Shenzhen, China) with primers listed in Table S2.

### Sequencing, assembly, annotation, and genome comparisons

Bacterial DNA was isolated using a DNA extraction kit (Axygen, Hangzhou, China). The genome of strain S3-4 was sequenced by Personalbio (Shanghai, China) using an Illumina Miseq system. A combination of three libraries containing 450 bp (paired-end), 3 kb and 8 kb (mate-paired) inserts was sequenced. The generated sequences were assembled using Newbler de novo (version 2.6)^32^, and the pre-assembled contigs were scaffolded using the SSPACE program^33^. Gaps between contigs were closed using GapCloser software (version 1.12; http://soap.genomics.org.cn) and PCR amplification. Prediction of open reading frames (ORFs) was accomplished using Glimmer 3.0 (http://www.cbcb.umd.edu/software/glimmer/), whereas RNAmmer 1.2^34^ and tRNAscan-SE (Version 1.3.1)^35^ were used for the identification of rRNA and tRNA. Functional annotation of genes was done using BLAST2GO software^36^ and the refseq-protein database (https://www.ncbi.nlm.nih.gov/refseq/). A putative function was assigned to each gene using a cutoff E-value of ≤1 E^−06^. Predicted protein sequences of strains with and without DON-degradation activity, *Devosia* sp. 17-2-E-8^25^ and *Sphingobium japonicum* UT26^21^, respectively, were compared with strain S3-4 using bidirectional BLASTp comparisons with an E value cutoff of 10^−5^ as previously described^37^.

### Cloning, expression and purification of recombinant protein in *E. coli*

The *AKR18A1* gene was amplified by PCR from genomic DNA of strain S3-4 using KOD-plus-DNA polymerase (Toyobo, Shanghai, China) with the primers listed in Table S2. The PCR products were cloned into a pET-22b vector (Novagen, CA, USA) and transformed into *E. coli* BL21 for expression as a His_6_-fusion protein. Recombinant AKR18A1 protein was purified by affinity-chromatography and analyzed by SDS-PAGE as previously described^38^.

### Enzyme assays

Purified AKR18A1 protein (6 μg) was assayed for DON-degrading activity in a 50 μL mixture containing DON (100 μM) and 2 mM NADP^+^ at different temperatures. The reaction was terminated by adding an equal volume of methanol. The initial rate of substrate decrease was quantified by HPLC. Activity at different pH values was assayed using 50 mM solutions of the following buffers: sodium phosphate (pH 6.0–7.0), Tris-HCl (pH 7.0–9.0), and glycine NaOH (pH 9.0–11.0). Optimum temperatures were determined by assaying activity at temperatures ranging from 10 °C to 60 °C in 50 mM glycine NaOH buffer (pH 10.6).

Enzymatic kinetics of the AKR18A1 with each substrate and cofactor were measured with 6 μg of recombinant AKR18A1 enzyme in a final volume of 50 μL. For the oxidation reaction, the kinetics were determined in the glycine-NaOH buffer (pH 10.6). The DON concentrations in the assay ranged from 10 to 1500 µM, using an NADP^+^ concentration of 2 mM. For the reduction reaction, the kinetics were measured in sodium phosphate buffer (pH 7.0). The 3-oxo-DON concentration ranged from 50 to 1000 µM were used for the assays, with an NADH concentration of 0.2 mM. The reaction was incubated for 30 min at 45 °C. Reactions were stopped and the decrease in substrate quantity was quantified by HPLC analysis of the resultant mixture. Michaelis–Menten constants (Km and Vmax) were calculated with Origin 8 (Origin Software, CA).

### Disruption of an *AKR18A1* gene in S3-4

The ~850 bp upstream and downstream regions flanking the *AKR18A1* gene were PCR-amplified using primers listed in Table S2 and joined by PCR overlap extension. The resulting sequence was cloned into a suicide plasmid pK18*mobsacB* ^39^. This plasmid was introduced into S3-4 via conjugal transfer, and the mutant strain was identified as described previously^40^ with the primers listed in Table S2.

### ZEN catabolism by strain S3-4 and recombinant AKR18A1 protein

Strain S3-4 was assayed for ZEN, α-ZOL and β-ZOL catabolic activity in 1 mL of MM containing 100 μM of each substrate. Substrate concentration was determined by HPLC at 72 hai. For the recombinant AKR18A1 protein, assays of ZEN, α-ZOL and β-ZOL catabolism were performed in a 50 μL mixture containing 100 μM each substrate, 0.2 mM NADH or 2 mM NADP^+^ and 6 μg of purified protein. The reaction was terminated by adding an equal volume of methanol to the mixture, and substrate concentration was determined by HPLC.

### Glyoxal and methylglyoxal catabolism by recombinant AKR18A1 in an *E. coli* strain

The tolerance of the recombinant *E. coli* strain to GO and MG was determined as previously described^41^. *E. coli* BL21-(DE3) cells harbouring the pET22b::AKR18A1 expression construct or control pET22b vector were grown at 37 °C in LB liquid medium to early exponential phase (OD_600_ = 0.2), and the expression of His-AKR18A1 fusion protein was induced by the addition of 0.2 mM IPTG. After 60 min 10 mL aliquots were exposed to a 2 mM concentration of GO or MG, and OD_600_ was determined over the course of 4 h.

### GO and MG catabolism by recombinant AKR18A1 protein

The GO and MG catabolic activity of AKR18A1 was measured in a 50 μL mixture containing 100 μM each substrate, 0.2 mM NADPH and 6 μg purified protein. The reaction was terminated as described above, and substrate concentration was determined by HPLC as previously described^42^.

### Statistical analysis

All assays were performed in triplicate. The results were analyzed using ANOVA for multiple comparisons followed by the Duncan test using SAS software v.8.1 (SAS institute, Cary, NC, USA), with significance levels of 0.01.

## Electronic supplementary material

Supplementary Information
