# Supplementary material for: An aldo-keto reductase is responsible for Fusarium toxin-degrading activity in a soil Sphingomonas strain
Source: Sci Rep. 2017 Aug 25;7:9549. doi: 10.1038/s41598-017-08799-w (PMC5573404; doi:10.1038/s41598-017-08799-w)
Supplement: Supplementary file 1 — Supplementary Information [file 41598_2017_8799_MOESM1_ESM.pdf]

An aldo-keto reductase is responsible for *Fusarium* toxin-degrading activity in a soil *Sphingomonas* strain

Wei-Jie He[1,2], Limin Zhang[3], Shu-Yuan Yi[1,4], Xue-Ling Tang[1,4], Qing-Song Yuan[1,4], Mao-Wei Guo[1,4], Ai-Bo Wu[1,5], Bo Qu[1,4], He-Ping Li[1,2] & Yu-Cai Liao[1,4,6,*]

[1]Molecular Biotechnology Laboratory of Triticeae Crops, Huazhong Agricultural University, Wuhan 430070, China

[2]College of Life Science and Technology, Huazhong Agricultural University, Wuhan 430070, China

[3]State Key Laboratory of Magnetic Resonance and Atomic and Molecular Physics, Wuhan Centre for Magnetic Resonance, Wuhan Institute of Physics and Mathematics, the Chinese Academy of Sciences, Wuhan 430071, China

[4]College of Plant Science and Technology, Huazhong Agricultural University, Wuhan 430070, China

[5]Key Laboratory of Food Safety Research Institute for Nutritional Sciences, Shanghai Institutes for Biological Sciences, Chinese Academy of Sciences, Shanghai 200031, China

[6]National Center of Plant Gene Research (Wuhan), Huazhong Agricultural University, Wuhan 430070, China

*Correspondence: Yu-Cai Liao; Email: yucailiao@mail.hzau.edu.cn (Y.C.L)

Tel./Fax: +86-27-8728-3008

Legends to Supplementary Figures and Tables

Fig. S1. Phylogenetic tree based on partial 16S rDNA sequences of strain S3-4 and related microorganisms. The 16S rDNA sequence of strain S3-4 (1,445 bp) is 97% identical to that of *Sphingomonas leidyi* DSM 4733. The tree was constructed by the maximum-likelihood method. The GenBank accession numbers of the sequences are shown in parentheses. *Sinorhizobiumme liloti* 1021 was used as an outgroup. The bar indicates 1% sequence divergence.

Fig. S2. The derivatized structures of DON and its metabolites. A. Derivatized structure of DON with three TMS groups; B. Derivatized structure of 3-oxo-DON with two TMS groups; C. Derivatized structure of 3-epi-DON with three TMS groups. TMS, tetramethyl silane, [-Si(CH_3)$_3$].

Fig. S3. ^{1}H NMR spectra of compounds metabolized from DON by strain S3-4 (in $CDCl_3$ at 30℃). A. NMR spectra of compound A. B. NMR spectra of compound B. Structures of compound A and compound B are shown in the left insert of each figure.

Fig. S4. Pulsed-field gel electrophoresis of DNA inserts from BAC clones randomly selected from a BAC library constructed with S3-4 genomic DNA.

Fig. S5. Alignment of amino acid sequences of S3-4 AKR18A1 and other AKR members from bacteria using the PROMALS3D multiple sequence and structure alignment server (prodata.swmed.edu/ promals3d/). Representative sequences are colored according to predicted secondary structures (red: alpha-helix, blue: beta-strand). Consensus predicted secondary structure symbols: alpha-helix, h; beta-strand, e. Consensus amino acid symbols: conserved amino acids are represented by bold and uppercase letters; aliphatic (I, V, L): l, aromatic (Y, H, W, F): @, hydrophobic (W, F, Y, M, L, I, V, A, C, T, H): h, alcohol (S, T): o, polar residues (D, E,

H, K, N, Q, R, S, T): p, tiny (A, G, C, S): t, small (A, G, C, S, V, N, D, T, P): s, bulky residues (E, F, I, K, L, M, Q, R, W, Y): b, positively charged (K, R, H): +, negatively charged (D, E): -, charged (D, E, K, R, H): c. The catalytically active tetrad (Asp-57, Tyr-62, Lys-90, and His-131) is indicated by a gray background.

Fig. S6. Phylogenetic relationship of S3-4 AKR18A1 and other bacterial AKRs. Trees were constructed by the maximum likelihood method. GenBank accession numbers are given in brackets. The bars indicate 0.1 substitutions per amino acid position.

Fig. S7. HPLC profile of DON and product from reverse reaction of 3-oxo-DON by recombinant AKR18A1 in the presence of cofactor NADH. The profile in top panel showed the sample without AKR18A1. The profile in bottom panel showed the sample with AKR18A1.

Fig. S8. PCR analysis of the *AKR18A1* gene in the wild-type strain S3-4 and a mutant strain with a disrupted *AKR18A1* gene. Lane 1, control with no DNA template; Lanes 2 to 8, independent clones from the mutant strain (1,670 bp product); Lane 9, wild-type strain S3-4 (2,364 bp product).

Fig. S9. Growth and degradation efficiency of the wild-type strain S3-4 and the mutant strain *Δ*akr18a1 in mineral salts medium containing DON (100 μg/mL). DON concentration (filled symbols) and the amount of bacterial growth (open symbols) in S3-4 (triangles) and *Δ*akr18a1 (circles) were determined every 12 h for 72 h. The values given are the means of three biological replicates. The error bars represent the standard deviation.

Fig. S10. Catabolism of zearalenone (ZEN), α-zearalenol (α-ZOL) and β-zearalenol (β-ZOL) by strain S3-4 and recombinant AKR18A1 protein. A. HPLC profiles of ZEN catabolism by recombinant AKR18A1 in vitro in the presence of cofactor NADH. Insert: structures of ZEN, α-ZOL and β-ZOL. B. HPLC profiles of ZEN catabolism by recombinant AKR18A1 in vitro in the presence of cofactor NADPH. C. HPLC profiles of recombinant AKR18A1 catalysis of α-ZOL oxidation in vitro in the presence of cofactor $NADP^+$. D. HPLC profiles of recombinant AKR18A1 catalysis of β-ZOL oxidation in vitro in the presence of cofactor $NADP^+$. E. HPLC profiles of α-ZOL in strain S3-4. F. HPLC profiles of strain S3-4 catalysis of β-ZOL oxidation. Red line: samples with recombinant AKR18A1 protein or S3-4, Black line: samples without recombinant AKR18A1 protein or S3-4.

Fig. S11. Degradation of glyoxal (GO) and methylglyoxal (MG) by recombinant AKR18A1 protein. In vivo protective effect of AKR18A1 against GO (A) and MG (B) toxicity in *E.coli*. BL21 (DE3) cells contained either the control plasmid pET-22b (black line) or the AKR18A1 expression plasmid (red line). Degradation of GO (C) and MG (D) by recombinant AKR18A1 protein. Insert: structures of GO and MG. Red line: samples with recombinant AKR18A1 protein, Black line: samples without recombinant AKR18A1 protein.

Table S1. ^{1}H NMR data for DON metabolites A and B (in $CDCl_3$ at 30℃).

Table S2. Primers used in this study.

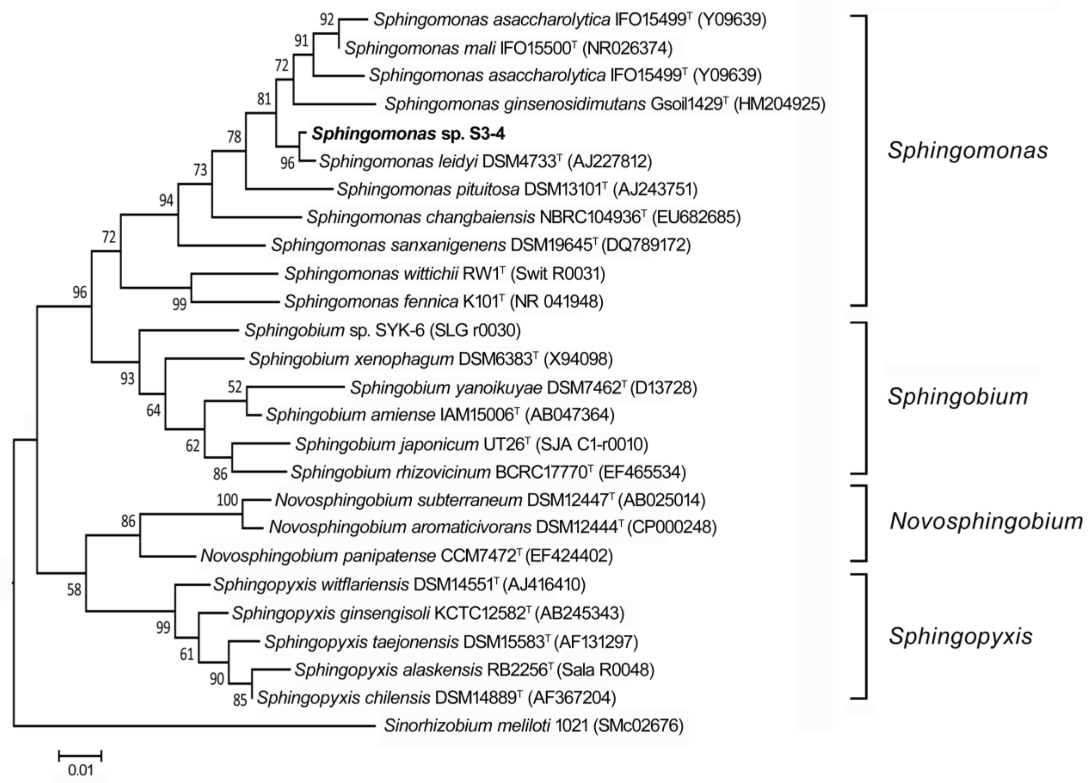

Sphingomonas asaccharolytica IFO15499T (Y09639)
Sphingomonas mali IFO15500T (NR026374)
Sphingomonas asaccharolytica IFO15499T (Y09639)
Sphingomonas ginsenosidimutans Gsoil1429T (HM204925)
Sphingomonas sp. S3-4
Sphingomonas leidyi DSM4733T (AJ227812)
Sphingomonas pituitosa DSM13101T (AJ243751)
Sphingomonas changbaiensis NBRC104936T (EU682685)
Sphingomonas sanxanigenens DSM19645T (DQ789172)
Sphingomonas wittichii RW1T (Swit R0031)
Sphingomonas fennica K101T (NR 041948)
Sphingobium sp. SYK-6 (SLG r0030)
Sphingobium xenophagum DSM6383T (X94098)
Sphingobium yanoikuyae DSM7462T (D13728)
Sphingobium amiense IAM15006T (AB047364)
Sphingobium japonicum UT26T (SJA C1-r0010)
Sphingobium rhizovicinum BCRC17770T (EF465534)
Novosphingobium subterraneum DSM12447T (AB025014)
Novosphingobium aromaticivorans DSM12444T (CP000248)
Novosphingobium panipatense CCM7472T (EF424402)
Sphingopyxis witflariensis DSM14551T (AJ416410)
Sphingopyxis ginsengisoli KCTC12582T (AB245343)
Sphingopyxis taejonensis DSM15583T (AF131297)
Sphingopyxis alaskensis RB2256T (Sala R0048)
Sphingopyxis chilensis DSM14889T (AF367204)
Sinorhizobium meliloti 1021 (SMc02676)
Sphingomonas
Sphingobium
Novosphingobium
Sphingopyxis
0.01

Figure S1

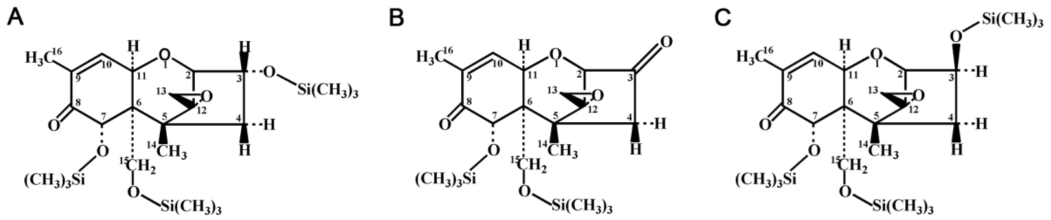

DON-TMS   3-oxo-DON-TMS   3-epi-DON-TMS

Figure S2

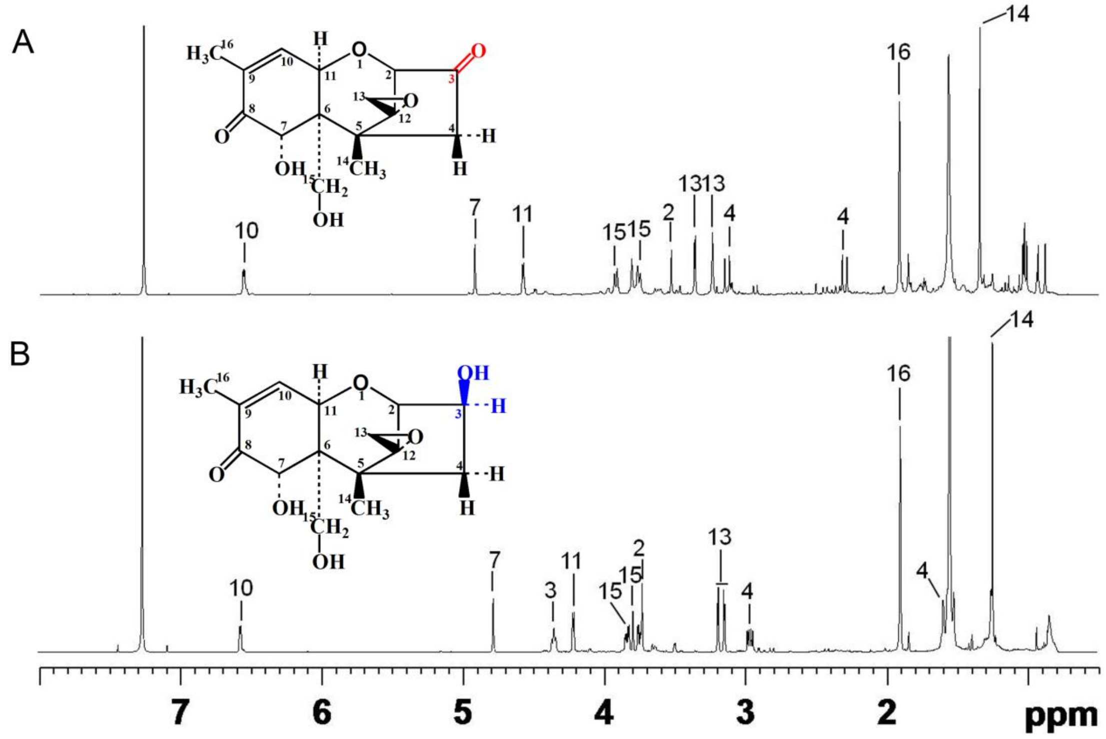

Figure S3

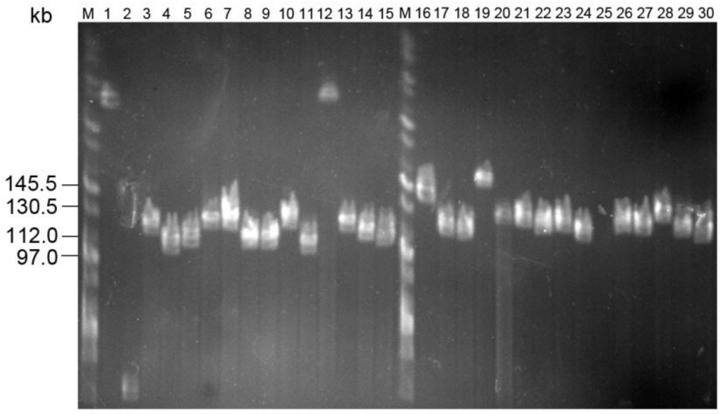
kb
M 1 2 3 4 5 6 7 8 9 10 11 12 13 14 15 M 16 17 18 19 20 21 22 23 24 25 26 27 28 29 30
145.5
130.5
112.0
97.0

Figure S4

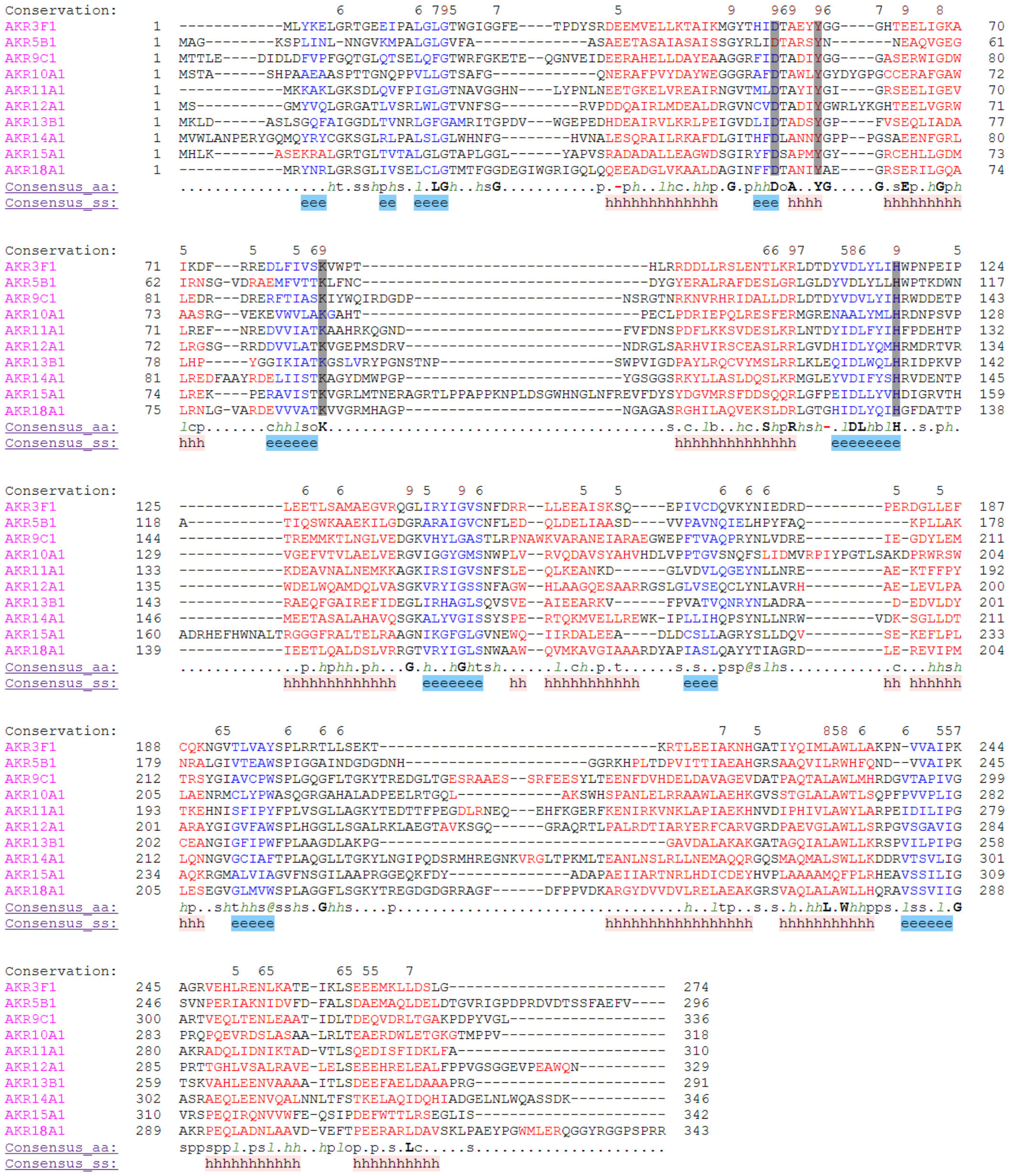

Figure S5

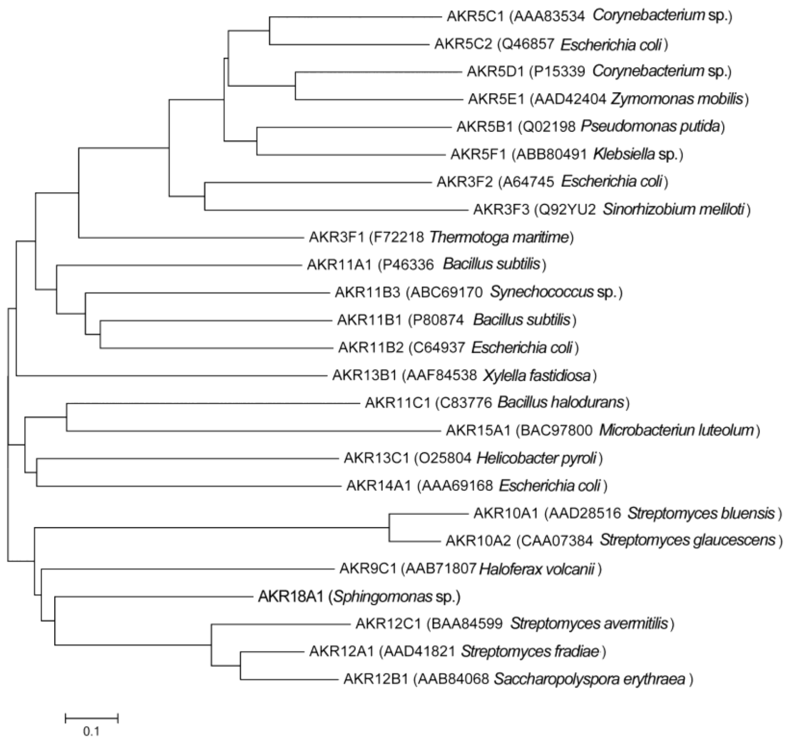
AKR5C1 (AAA83534 Corynebacterium sp.)
AKR5C2 (Q46857 Escherichia coli)
AKR5D1 (P15339 Corynebacterium sp.)
AKR5E1 (AAD42404 Zymomonas mobilis)
AKR5B1 (Q02198 Pseudomonas putida)
AKR5F1 (ABB80491 Klebsiella sp.)
AKR3F2 (A64745 Escherichia coli)
AKR3F3 (Q92YU2 Sinorhizobium meliloti)
AKR3F1 (F72218 Thermotoga maritime)
AKR11A1 (P46336 Bacillus subtilis)
AKR11B3 (ABC69170 Synechococcus sp.)
AKR11B1 (P80874 Bacillus subtilis)
AKR11B2 (C64937 Escherichia coli)
AKR13B1 (AAF84538 Xylella fastidiosa)
AKR11C1 (C83776 Bacillus halodurans)
AKR15A1 (BAC97800 Microbacterium luteolum)
AKR13C1 (O25804 Helicobacter pyroli)
AKR14A1 (AAA69168 Escherichia coli)
AKR10A1 (AAD28516 Streptomyces bluensis)
AKR10A2 (CAA07384 Streptomyces glaucescens)
AKR9C1 (AAB71807 Haloferax volcanii)
AKR18A1 (Sphingomonas sp.)
AKR12C1 (BAA84599 Streptomyces avermitilis)
AKR12A1 (AAD41821 Streptomyces fradiae)
AKR12B1 (AAB84068 Saccharopolyspora erythraea)
0.1

Figure S6

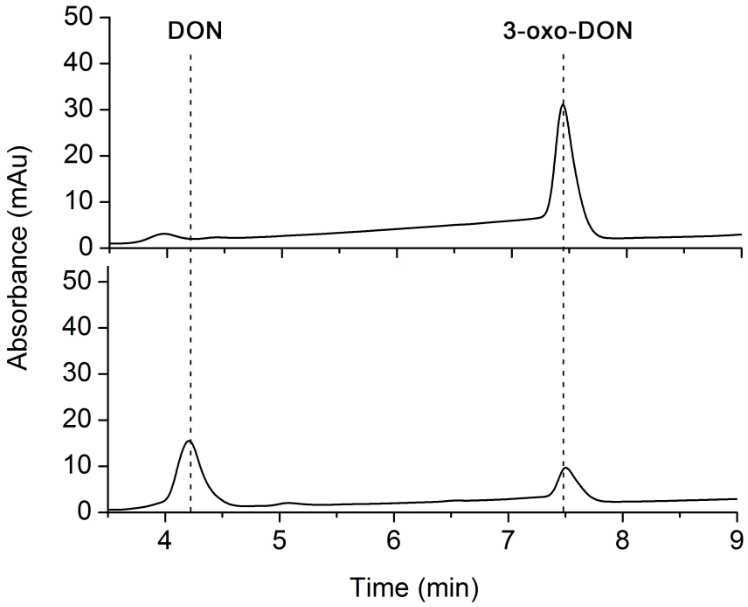

Figure S7

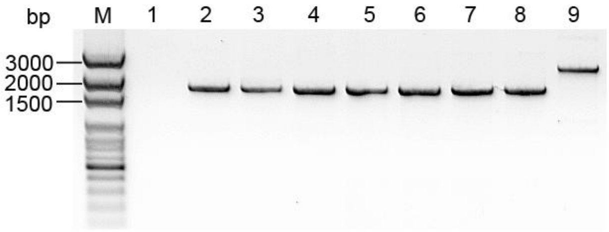

Figure S8

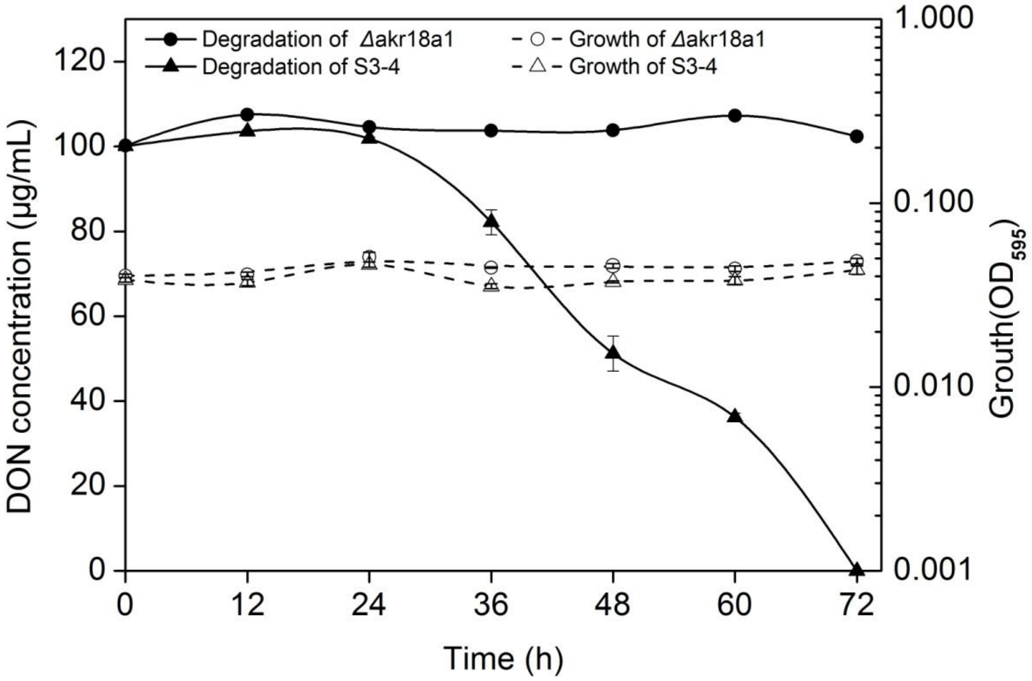
Degradation of Δakr18a1
Degradation of S3-4
Growth of Δakr18a1
Growth of S3-4
DON concentration (µg/mL)
Grouth(OD_{595})
Time (h)
0
20
40
60
80
100
120
0
12
24
36
48
60
72
0.001
0.010
0.100
1.000

Figure S9

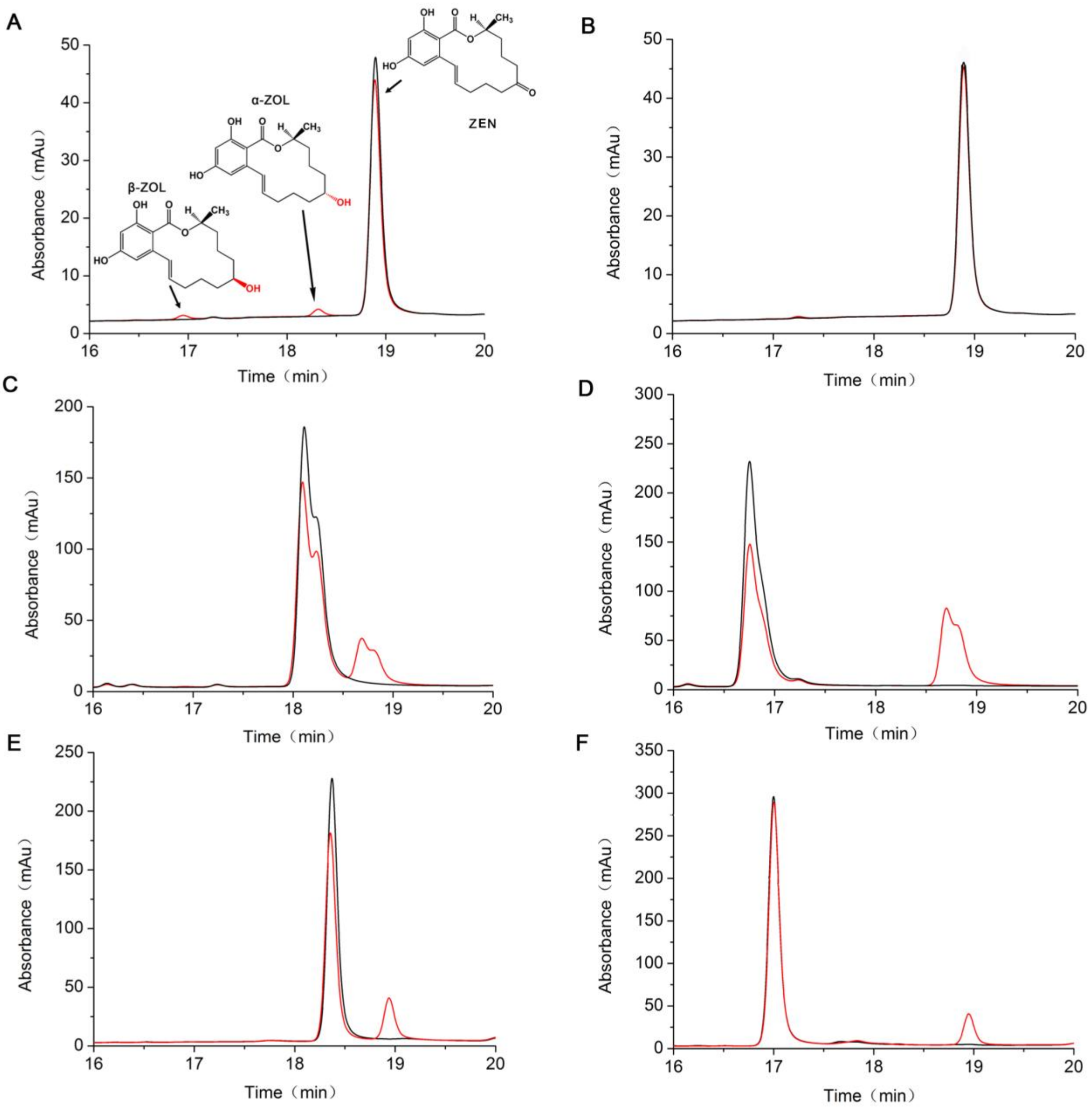

Figure S10

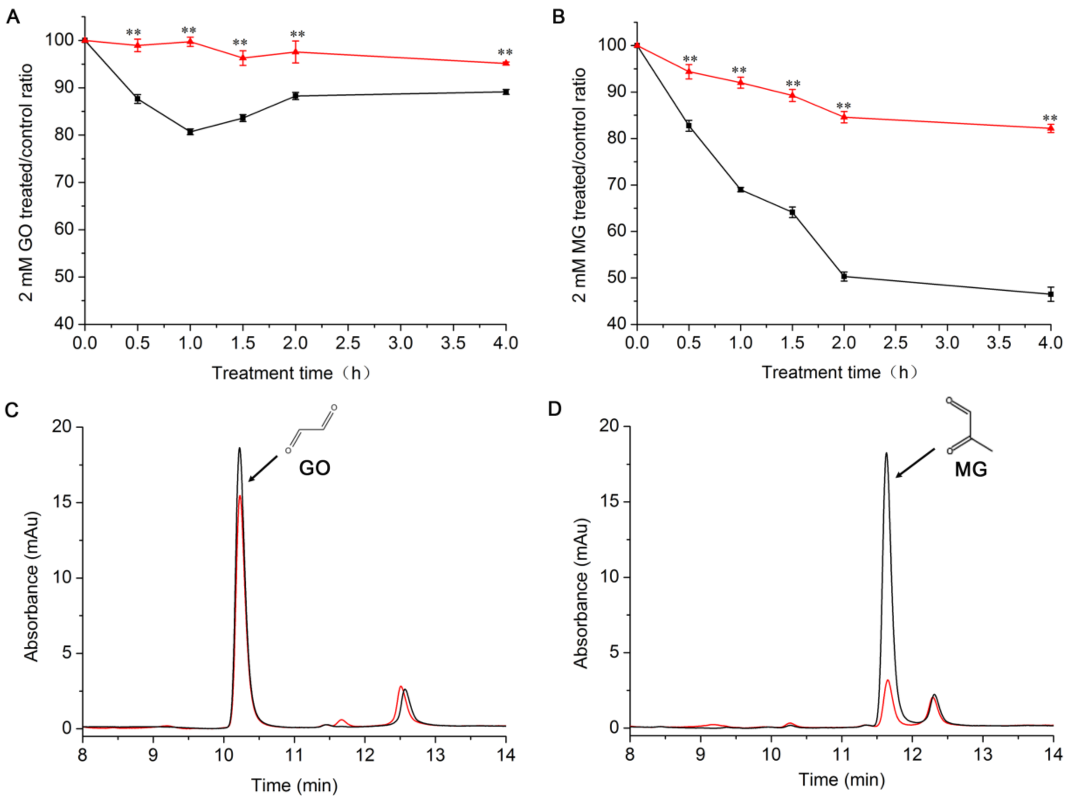
A
B
C
D
2 mM GO treated/control ratio
2 mM MG treated/control ratio
Treatment time（h）
Treatment time（h）
Absorbance (mAu)
Absorbance (mAu)
Time (min)
Time (min)
GO
MG
**

Figure S11

Table S1. ^{1}H NMR data for DON metabolites A and B (in CDCl3 at 30℃)

Position	^{1}H-NMR δ_H, ppm (multiplicity, *J* in Hz)			
	Metabolite A	3-oxo-DON	Metabolite B	3-epi-DON
2	3.52 (s)	3.52 (s)	3.70 (s)	3.70 (s)
3	-	-	4.32 (d, 3.0, 7.5)	4.33 (dd, 3.0, 7.5)
4	3.13 (d, 18.0)<br>2.26 (dd, 4.0, 15.0)	3.13 (d, 18)<br>2.28(br d,19)	2.92 (dd,7.5, 15.5)<br>1.60 (dd,3.0, 15.5)	2.93 (dd, 7.5, 15.5)<br>1.60(dd, 3.0, 15.5)
5	-	-	-	-
6	-	-	-	-
7	4.91 (s)	4.90 (s)	4.74 (s)	4.75 (s)
8	-	-	-	-
9	-	-	-	-
10	6.55 (d, 6.0)	6.55 (br d, 6.0)	6.53 (d, 6.0)	6.53 (dd, 1.5, 6.0)
11	4.57 (d, 5.6)	4.57 (br d, 6.0)	4.21 (d, 6.0)	4.19 (d, 6.0)
12	-	-	-	-
13	3.36 (d, 4.0)<br>3.23 (d, 4.0)	3.35 (d, 4)<br>3.22 (d, 4)	3.14 (d, 4.5)<br>3.12 (d, 4.5)	3.16 (d, 4.5)<br>3.11 (d, 4.5)
14	1.33 (s)	1.33 (3H,s)	1.22 (s)	1.22 (3H, s)
15	3.91 (d, 12.0)<br>3.74 (d, 12.0)	3.90 (d, 12.0)<br>3.74 (d, 12.0)	3.80 (d, 12.0)<br>3.73 (d, 12.0)	3.80 (d, 12.0)<br>3.72 (d, 12.0)
16	1.92 (s)	1.90 (3H, br s)	1.88 (s)	1.87 (3H, s)

Table S2. Primers used for PCR analysis

Gene	Sequence (5' to 3')
P450	Forward: TGGCATCAAAGTGACCGAAGG
	Reverse: GGCCCACTGGAGAAAGACAAT
221	Forward: CCATAAGCACCCGTGTTTCA
	Reverse: GCACCGAGATCATAATAAGT
C12	Forward: CTCCCTCCTACCCACTGACC
	Reverse: TCGCTTCTGTCTTATCCTCC
C7	Forward: GTGCCTTTGTTCACAGAGTT
	Reverse: GTTTTGCAGCTTCCAGTTAG
C14	Forward: GCTGTGGGTGTTCGGATGTC
	Reverse: TTTCCCTTCCTTCTTTACGG
β-actin	Forward: GCTGTTCCAGCCATCTCATGT
	Reverse: CGATCAGCAATTCCAGGAAAC
BAC end sequencing	Forward:AACGACGGCCAGTGAATTG
	Reverse: GATAACAATTTCACACAGG
AKR18A1	Forward: GGAATTCGATGCGCTACAACCGGCTCGGCCG
	Reverse: CCCAAGCTTGCGCCGCGGCGACGGGCCG
AKR18A1 upstream	Forward: GGAATTCCGTCACTTCGCGGGCGATGGTC
	Reverse: CCTCGGGCGTGAACTCTACGCCAGGATCCGCTCCGAACGACC
AKR18A1 downstream	Forward: CGTAGAGTTCACGCCCGAGGAG
	Reverse: CCCAAGCTTCGATGTGCTGTCCCGCTGTCTC
